# TGF-β-induced activation of conjunctival fibroblasts is modulated by FGF-2 and substratum stiffness

**DOI:** 10.1371/journal.pone.0242626

**Published:** 2020-11-18

**Authors:** Tomoyo Matsumura, Tomokazu Fujimoto, Akiko Futakuchi, Yuji Takihara, Fumika Watanabe-Kitamura, Eri Takahashi, Miyuki Inoue-Mochita, Hidenobu Tanihara, Toshihiro Inoue

**Affiliations:** 1 Department of Ophthalmology, Faculty of Life Sciences, Kumamoto University, Kumamoto, Japan; 2 Department of Medical Cell Biology, Institute of Molecular Embryology and Genetics, Kumamoto University, Kumamoto, Japan; 3 Director of Hospital, Kumamoto University Hospital, Kumamoto, Japan; Chang Gung University, TAIWAN

## Abstract

**Purpose:**

This study aimed to investigate the effects of substratum stiffness on the sensitivity of human conjunctival fibroblasts to transforming growth factor (TGF)-β, and to explore the molecular mechanism of action.

**Methods:**

Human conjunctival fibroblasts were cultured on collagen-coated plastic or silicone plates. The stiffness of the silicone plates was 0.2 or 64 kPa. Cells were treated by 2.5 ng/mL TGF-β2 with or without fibroblast growth factor (FGF)-2 (0–100 ng/mL) for 24 h or 48 h. The protein expression levels were determined by Western blot analysis. Cell proliferation was assessed using the WST-8 assay.

**Results:**

FGF-2 suppressed the TGF-β-induced expression of α-smooth muscle actin (SMA) and collagen type I (Col I), but not fibronectin (FN). Both FGF-2 and TGF-β2 increased cell proliferation without an additive effect. The induction of α-SMA by TGF-β2 was decreased on the soft substratum, without any change in the expression level or subcellular location of Yes-associated protein/transcriptional coactivator with PDZ-binding motif (YAP/TAZ). FGF-2 suppressed TGF-β-induced α-SMA expression even on the soft substratum.

**Conclusions:**

FGF-2 treatment and a soft substratum suppressed TGF-β-induced transdifferentiation of conjunctival fibroblasts into myofibroblasts. FGF-2 attenuated the TGF-β-induced expression of α-SMA, even on a soft substratum.

## Introduction

Wound healing mechanisms in the conjunctiva are involved in the pathology of various ocular diseases, and in the outcomes of ocular surgeries, including for pterygium and glaucoma [1–5]. Among the numerous cell types that contribute to wound closure, fibroblasts have been known to play a particularly important role by transdifferentiation into myofibroblasts. Because myofibroblasts possess higher contractibility, higher cell proliferation, and produce more extracellular matrix compared to naive fibroblasts, excessive activity of myofibroblasts results in the formation of hard scar tissue, which can affect the ocular surface and outcomes of surgery. It is therefore important to identify the factors that control conjunctival fibroblasts, to maintain the ideal ocular surface conditions and promote good vision [6–8].

The surgical results of trabeculectomy, a representative type of glaucoma filtration surgery, depend on the extent of postoperative scarring at the surgical site. Excessive wound healing in response to surgical trauma may cause surgical failure; previous ocular surgery is a well-known risk factor for trabeculectomy failure. Conjunctival scarring at the operative site may adversely affect subsequent trabeculectomy, not only because of technical difficulties encountered during surgery but also because preexisting conjunctival fibrosis may exaggerate postoperative scarring [8,9]. Although clinicians report that conjunctival tissue becomes stiff after surgery, it remains unclear whether such stiffness directly compromises trabeculectomy outcomes. We hypothesized that prior ocular surgeries compromise trabeculectomy outcomes by creating a hard substratum for conjunctival fibroblasts, thus enhancing their transdifferentiation into myofibroblasts.

Yes-associated protein (YAP) and transcriptional coactivator with PDZ-binding motif (TAZ) are involved in the Hippo pathway. YAP/TAZ are key transducers of mechanical stress, regulated by the stabilities and intracellular locations of the proteins [10,11]. In the absence of mechanical stress, the pathway is activated and YAP/TAZ are destabilized and degraded. In contrast, the kinase cascade is inactivated under mechanical stress, and stabilized YAP/TAZ are translocated to the nucleus. YAP/TAZ subsequently interact with TEAD transcription factors to enhance the transcription of genes involved in cell proliferation and survival, and fibrosis [12–14].

The multifunctional secreted protein fibroblast growth factor (FGF)-2, also known as basic FGF, is a member of the FGF family. During cutaneous wound healing, FGF-2 exhibits anti-scarring effects, reducing the number of myofibroblasts by antagonizing myofibroblast transdifferentiation and inducing apoptosis of myofibroblasts [15–17]. In addition, FGF-2 is secreted in response to mechanical stress, a factor that modulates wound healing in various tissue, such as cartilage, periodontal ligament, bone marrow, vascular smooth muscle, bladder smooth muscle, and skeletal muscle [18–27]. Although FGF-2 reportedly stimulates transforming growth factor (TGF)-β-mediated cell proliferation in subconjunctival fibroblasts directly [28], the relationships among TGF-β, FGF-2, and mechanical stress during conjunctival wound healing remain unclear. Furthermore, the cross-reaction between FGF-2 and soft substratum during transdifferentiation into myofibroblasts has not been investigated thoroughly, either in the eye or in other tissues/organs.

Here we report that FGF-2 inhibited TGF-β-induced transdifferentiation of conjunctival fibroblasts into myofibroblasts. Although FGF-2 and a soft substratum did not affect TAZ, which is a mechano-sensing molecule, TAZ induction by TGF-β, soft substratum, and FGF-2 additively suppressed TGF-β-induced α-smooth muscle actin (SMA) expression.

## Materials and methods

### Cells

Primary human conjunctival fibroblasts (cell line numbers 5965, 20258, and 3072; ScienCell Research Laboratories, Carlsbad, CA, USA) were cultured according to the manufacturer’s recommendations, and performed all experiments with three different cell lines, as reported previously [29]. Briefly, cells were cultured in Fibroblast Medium (ScienCell Research Laboratories) supplemented with 2% fetal bovine serum (FBS), fibroblast growth supplement (ScienCell Research Laboratories), and penicillin (100 U/mL)/streptomycin (100 μg/mL) solution (Complete FM; ScienCell Research Laboratories) on poly-L-lysine-coated culture dishes at 37°C in 5% CO_2_. Cells at passages 4–6 were used in all experiments and were passaged in a manner ensuring 60% confluence on the following day. For assessment of the effects of substratum stiffness, cells were plated on 6-well dishes or CytoSoft^®^ (Advanced BioMatrix, San Diego, CA, USA) coated with collagen type I (Col I) according to the manufacturer’s protocol. The cells were treated with 2.5 ng/mL recombinant human TGF-β2 (R&D Systems, Minneapolis, MN, USA), FGF-2 (R&D Systems), and 30 nM PD173074 (Tokyo Chemical Industry, Tokyo, Japan) for 24 or 48 h. The control groups, such as FGF-2 zero ng/mL, were treated by solvent only. Each cytokine or inhibitor was added one after another for combination treatments. We checked each cell strain in terms of fibroblast morphology and expression of a fibroblast marker (fibroblast-specific protein 1 [FSP1], also known as S100A4). We confirmed that all three cell lines did not undergo morphological changes during passage. The stiffness of CytoSoft^®^ was validated by the manufacturer. According to the data sheet, the gel surfaces of CytoSoft^®^ products are functionalized to form covalent bonds with amines located on proteins. The functionalization is stable and the reaction does not require a catalyst, facilitating coating of the gel surfaces with matrix proteins and cell plates. The data sheet uses type I collagen as an example of a well coating; we used type I collagen in the present study. We also tested fibronectin, but the cells failed to adhere well.

### Western blotting

Western blotting was conducted as described previously, with minor modifications [29], using the following primary antibodies: anti-α-SMA (1:1,000; Sigma-Aldrich, St. Louis, MO, USA), anti-fibronectin (FN) (1:1,000; Abcam, Cambridge, UK), anti-Col I (1:1,000; Abcam), anti-YAP (1:1,00, cat. no. sc-101199; Santa Cruz Biotechnology, Dallas, TX, USA), anti-TAZ (1:1,000, cat. no. 8418; Cell Signaling Technology), and anti-S100A4 (1:1,000, cat. no. ab124805; Abcam). The secondary antibodies included horseradish peroxidase (HRP)-linked anti-rabbit (1:2,000; Cell Signaling Technology), HRP-linked anti-mouse (1:2,000; Cell Signaling Technology), and mouse anti-mouse (1:20,000; GE Healthcare, Chicago, IL, USA). The experiments were conducted in triplicate.

### Cell proliferation assay

Human conjunctival fibroblasts were seeded in 96-well plates at a density of 1 × 10^4^ cells per well. Cells were treated with 2.5 ng/mL TGF-β2 and/or increasing concentrations of FGF-2 (0–100 ng/mL) for 48 h. Proliferation was assessed using the WST-8 assay (Dojindo Laboratories, Kumamoto, Japan), which is based on the MTT assay but uses WST-8 [2-(2-methoxy-4-nitrophenyl)-3-(4-nitrophenyl)-5-(2,4-disulfophenyl)-2H-tetrazolium]. Each absorbance is proportional to the number of viable cells (Tominaga et al., 1999). The experiments were conducted in triplicate.

### Immunofluorescence assay

Immunofluorescence assay was conducted as described previously with minor changes [29]. Briefly, cells were washed twice with phosphate-buffered saline (PBS) and fixed with 4% (v/v) paraformaldehyde in PBS for 15 min at room temperature, and then washed with cytoskeletal buffer (10 mM 2-morpholinoethansulfonic acid potassium salt, 150 mM NaCl, 5 mM EGTA, 5mM MgCl_2_, and 5 mM glucose, pH 6.1) followed by serum buffer (10% [v/v] FBS and 0.2 mg/mL sodium azide in PBS). For permeabilization, cells were placed in 0.5% (v/v) Triton X-100 in PBS for 12 min at room temperature and then blocked with serum buffer at room temperature for 30 min. The cells were then incubated with anti-FSP1 (1:250; Abcam), anti-α-SMA antibodies (1:400; Sigma-Aldrich), anti-YAP antibodies (1:200; Santa Cruz Biotechnology), anti-TAZ antibodies (1:100; BD Pharmingen, San Jose, CA, USA), or anti-TEAD antibodies (1:100; Cell Signaling Technology) at 4°C overnight. After two washes in serum buffer, the cells were incubated with the anti-mouse IgG secondary antibody Alexa Fluor 488 (1:1,000; Thermo Fisher Scientific, Waltham, MA, USA), the anti-rabbit IgG secondary antibody Alexa Fluor 594 (1:1,000; Thermo Fisher Scientific), Alexa Fluor 546 phalloidin (1:200; Thermo Fisher Scientific), and/or DAPI solution (1:1,000; Dojindo Laboratories) at room temperature for 1 h. The cells were washed three times with PBS and observed under a vertical confocal microscope (FV1000MPE; Olympus, Tokyo, Japan). Nuclear localizations of YAP, TAZ, and TEAD were quantified as merged area with DAPI staining using MetaMorph^®^ (Ver. 7.8.8.0, Molecular Devices, CA, USA).

### Quantitative RT-PCR

RNA was isolated from the cells using NucleoSpin^®^ RNA (Takara Bio, Shiga, Japan), according to the manufacturer’s protocol. RNA was reverse transcribed using the PrimeScript RT-PCR kit (Takara Bio) and subjected to quantitative PCR (TB Green Premix Ex Taq II; Takara Bio) using a StepOnePlus Real-time PCR System (Thermo Fischer Scientific). All expression data were normalized to levels of the Glyceraldehyde-3-phosphate dehydrogenase (GAPDH) RNA. The primer sequences are listed in Table 1.

**Table 1:** Primer sequences in the quantitative RT-PCR.

Gene	Primer sequences (5’ to 3’)	Product size (bp)
*FN1 (fibronectin)*	F: CGGTGGCTGTCAGTCAAAG	130
	R: AAACCTCGGCTTCCTCCATAA	
*COL1A1*	F: GTGCGATGACGTGATCTGTGA	119
	R: CGGTGGTTTCTTGGTCGGT	
*ACTA2*	F: GTGTTGCCCCTGAAGAGCAT	109
	R: GCTGGGACATTGAAAGTCTCA	
*YAP1*	F: TAGCCCTGCGTAGCCAGTTA	170
	R: TCATGCTTAGTCCACTGTCTGT	
*WWTR1 (TAZ)*	F: TCCCAGCCAAATCTCGTGATG	122
	R: AGCGCATTGGGCATACTCAT	
*GAPDH*	F: GCACCGTCAAGGCTGAGAAC	138
	R: TGGTGAAGACGCCAGTGGA	

### Enzyme-linked immunosorbent assay (ELISA)

FGF-2 concentrations were measured using an ELISA kit (ab99979; Abcam) according to the manufacturer’s protocol. Briefly, 100 μL amounts of standard solutions or conditioned media (48 h after stimulation) were added to the wells of 96-well trays pre-coated with an anti-FGF-2 antibody and incubated overnight at 4°C with shaking. After washing, 100 μL of biotinylated FGF-detecting antibody was added to each well, followed by incubation for 1 h at room temperature with shaking. After washing, 100 μL of horseradish peroxidase-streptavidin solution was added to each well, followed by incubation for 45 min at room temperature with shaking. After washing, 100 μL of TMB One-Step Substrate Reagent was added to each well, followed by incubation for 30 min at room temperature in the dark with shaking. Finally, 50 μL of Stop Solution was added to each well and the absorbances at 450 nm were measured using a microplate reader (Multiskan FC; Thermo Fisher Scientific).

### Statistical analysis

The JMP V8 statistical software package (SAS Institute, Cary, NC, USA) was used to analyze the data. The two-way ANOVA test and the Tukey-Kramer comparison test were used for multiple comparisons. A p-value of less than 0.05 was indicative of statistical significance.

## Results

### Effects of FGF-2 on the transdifferentiation of conjunctival fibroblasts

**Fig 1:**
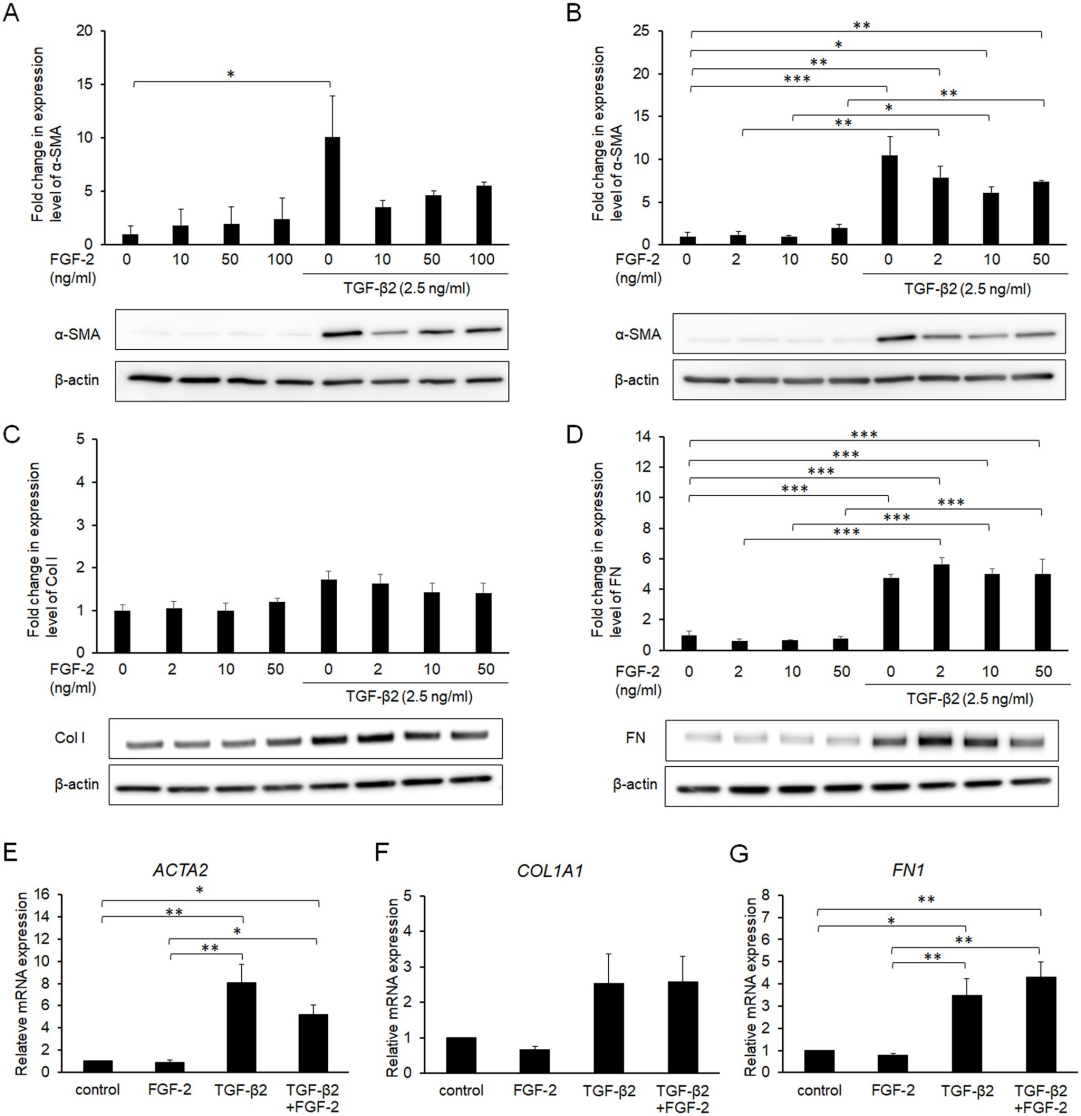
Effects of fibroblast growth factor (FGF)-2 on α-smooth muscle actin (SMA), collagen type I (Col I), and fibronectin (FN). Conjunctival fibroblasts were treated with FGF-2 (0–100 ng/mL) in the presence or absence of 2.5 ng/mL transforming growth factor (TGF)-β2 for 48 h, and subjected to Western blot analysis (A-D) or quantitative RT-PCR analysis (E-G). In the protein level, FGF-2 suppressed the TGF-β-induced expression of α-SMA (A, B) and Col I (C), but not FN (D). In the mRNA level, FGF-2 suppressed expression of only *ACTA2* coding α-SMA (E-G). Data are shown as the mean ± SE, n = 3.

**Fig 2:**
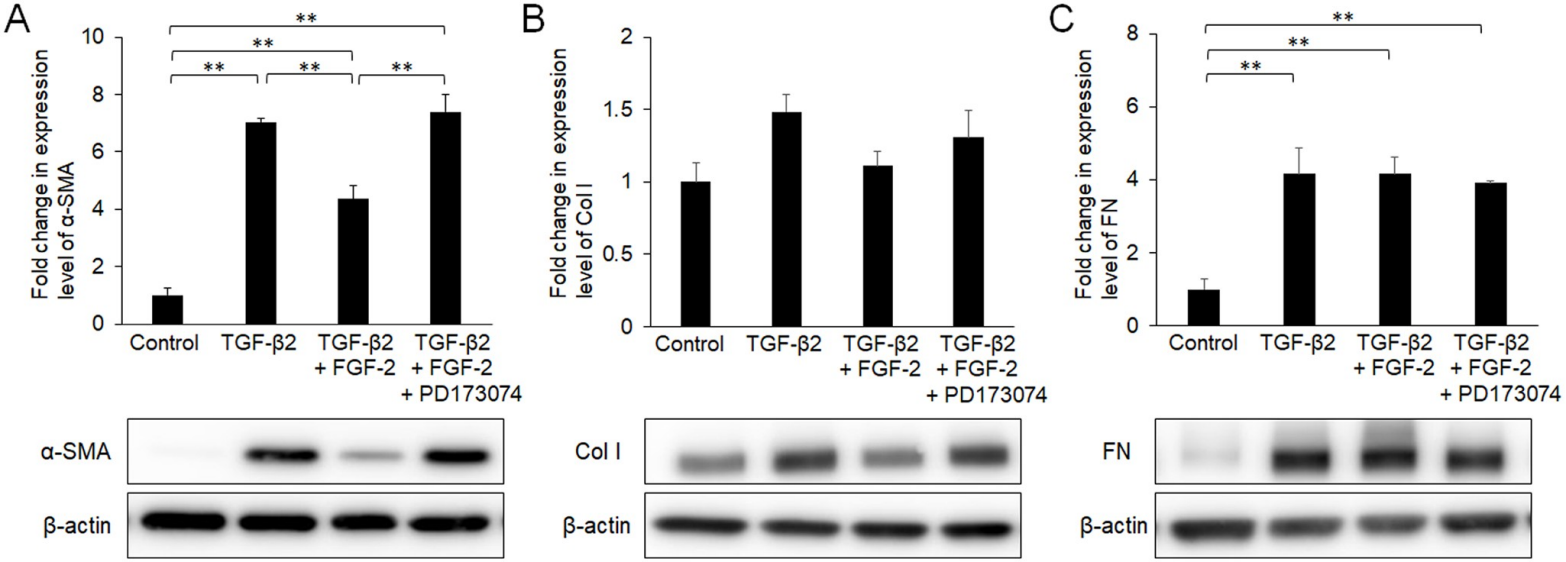
Effects of fibroblast growth factor (FGF)-2 and the FGF receptor inhibitor PD173074 on α-smooth muscle actin (SMA), collagen type I (Col I), and fibronectin (FN) expression. Conjunctival fibroblasts were treated with a combination of 10 ng/mL FGF-2, 30 nM PD173074, and 2.5 ng/mL transforming growth factor (TGF)-β2 for 48 h and subjected to Western blot analysis. The suppressive effect of FGF-2 on α-SMA (A) was diminished by PD173074, which did not affect Col I (B) or FN (C) significantly. **p < 0.01. Data are shown as the mean ± SE, n = 3.

Conjunctival fibroblasts were treated for 48 h with various doses (2, 10, 50, and 100 ng/mL) of FGF-2 in the presence or absence of TGF-β2. Every dose of FGF-2 tended to suppress α-SMA induction by TGF-β2, and the effect of 10 ng/mL FGF-2 was the greatest among the doses tested (Fig 1A and 1B). Although FGF-2 suppressed the TGF-β-induced expression of Col I to some extent (Fig 1C), while the effects of FGF-2 on the expression of fibronectin were limited (Fig 1D). FGF-2 did not affect the expression levels of these proteins in the absence of TGF-β2 (Fig 1A–1D). The effect of FGF-2 on α-SMA protein was similar with its effect on mRNA expression of ACTA2 that codes α-SMA (Fig 1E). Additionally, its effects on mRNAs that code Col I and fibronectin were not significant. FGF-2-mediated suppression of α-SMA expression was eliminated by the FGF receptor inhibitor PD173074 (Fig 2), suggesting that the suppressive effect was transduced via FGF receptors.

**Fig 3:**
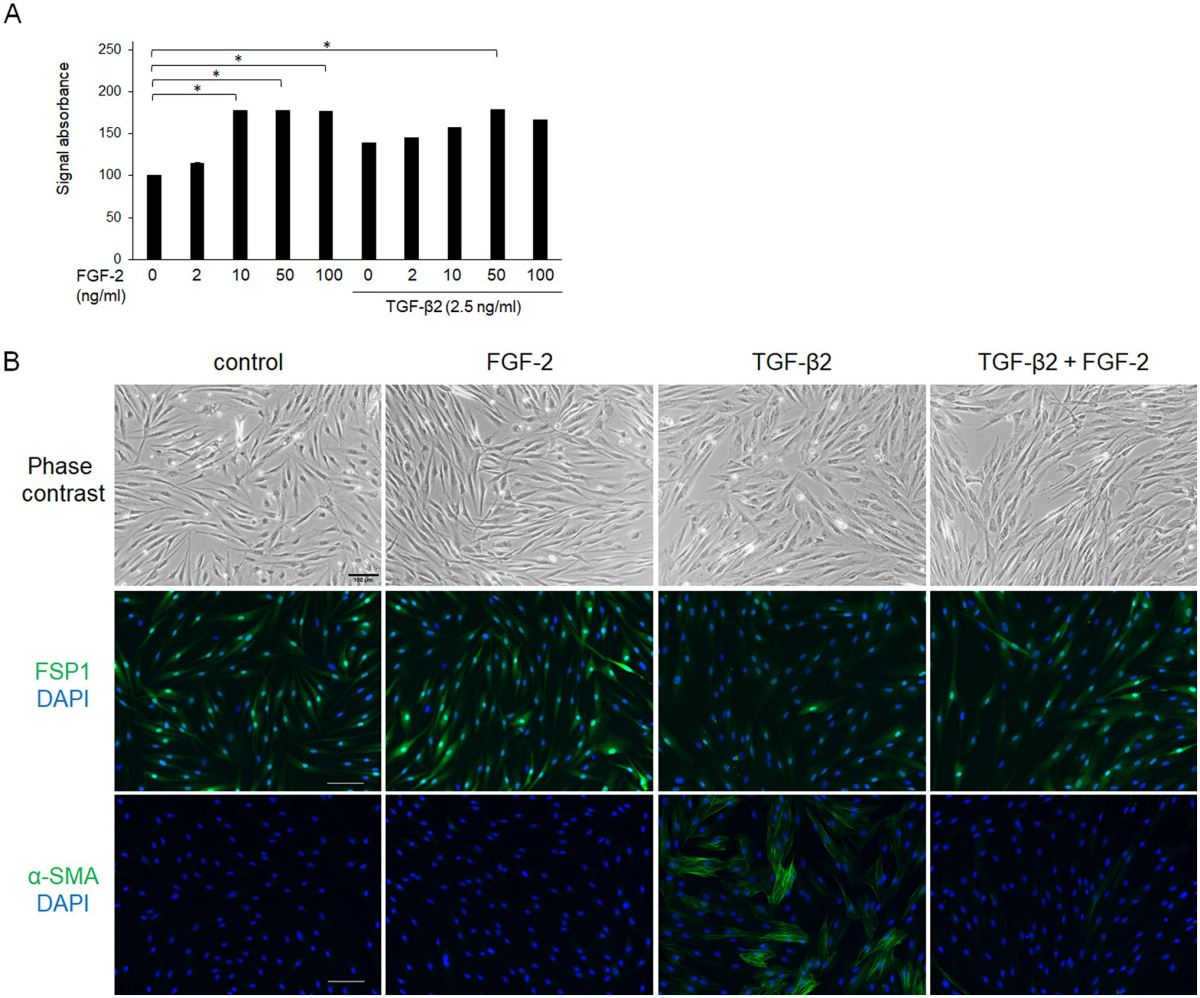
Effects of FGF-2 on cell proliferation (A), cell morphology, and expressions of cell-type markers (B). Conjunctival fibroblasts were treated with FGF-2 (0–100 ng/mL) in the presence or absence of 2.5 ng/mL TGF-β2 for 48 h. Subsequently, cells were observed by phase contrast microscopy, and were subjected to the WST-8 assay or immunofluorescence assay. Both FGF-2 and TGF-β2 increased cell proliferation without an additive effect (A). TGF-β2 treatment induced some changes in cell shape (from spindle- to cobblestone-like); FGF-2 did not affect cell shape in the presence or absence of TGF-β2 (B). At all conditions, cells were FSP-1 positive, while α-SMA expressed only in the cells treated with TGF-β alone. *p < 0.05. Data are shown as the mean ± SE, n = 8 for WST-8 assay, n = 3 for immunofluorescence assay. Scale bar: 200 μm.

Treatment with FGF-2 increased the proliferation of conjunctival fibroblasts at higher concentrations (Fig 3A). Although TGF-β2 alone tended to increase the proliferation, the difference was not statistically significant. There was no additive effect of those two factors with respect to cell proliferation. TGF-β2 treatment induced some changes in cell shape, from spindle- to cobblestone-like, and FGF-2 did not affect the cell shape in the presence or absence of TGF-β2 (Fig 3B). At all conditions, cells were FSP-1 positive, while α-SMA expressed only in the cells treated with TGF-β alone.

### Relationship between FGF-2 treatment and mechanical stress

**Fig 4:**
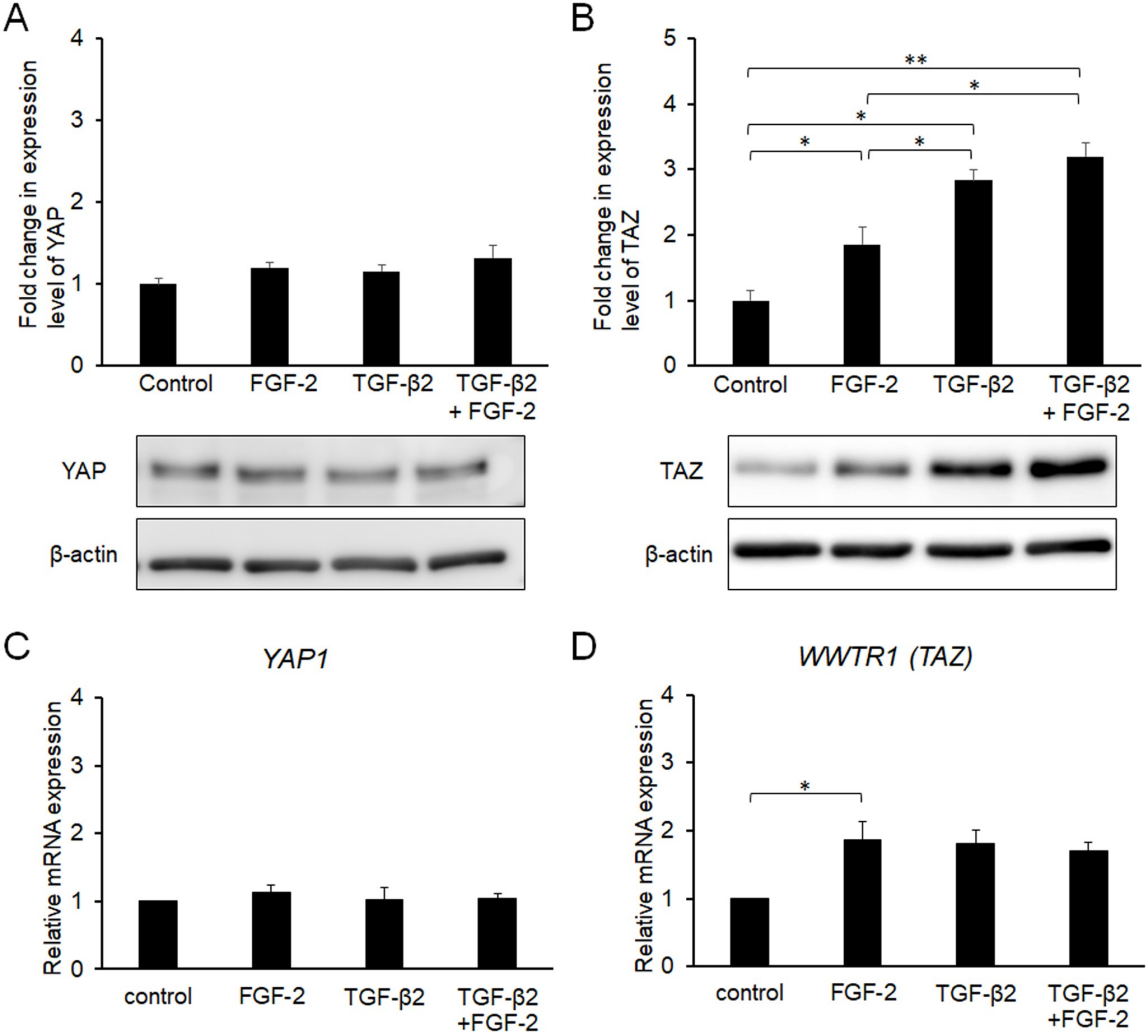
Effects of FGF-2 and/or TGF-β2 on the expression of YAP (A) and TAZ (B). Conjunctival fibroblasts were treated with FGF-2 (10 ng/mL) and/or TGF-β2 (2.5 ng/mL) for 24 h, and subjected to Western blot analysis (A, B) or quantitative RT-PCR analysis (C, D). Both FGF-2 and TGF-β2 increased the expression levels of YAP and TAZ. Although YAP expression was not significantly affected, FGF-2 increased the expression level of TAZ, and TGF-β2 enhanced this effect of FGF-2 in the protein level, but not in the mRNA level. *p < 0.05, **p < 0.01. Data are shown as the mean ± SE, n = 3.

Because FGF-2 responds to mechanical stress as described above, we evaluated the expression levels of YAP and TAZ, which are major mechano-transducers, after treatment with FGF-2 and/or TGF-β2 for 24 h. Although the effects of TGF-β2 on YAP expression were not significant, TGF-β2 increased the expression of TAZ (Fig 4A and 4B). Notably, FGF-2 also increased the expression level of TAZ, and TGF-β2 enhanced the effect of FGF-2. The effects of TGF-β2 or FGF-2 on mRNA expression coding YAP were not significant (Fig 4C) as well as the protein. Although both FGF-2 and TGF-β2 increased mRNA expressions coding TAZ, there was not additive effect (Fig 4D).

**Fig 5:**
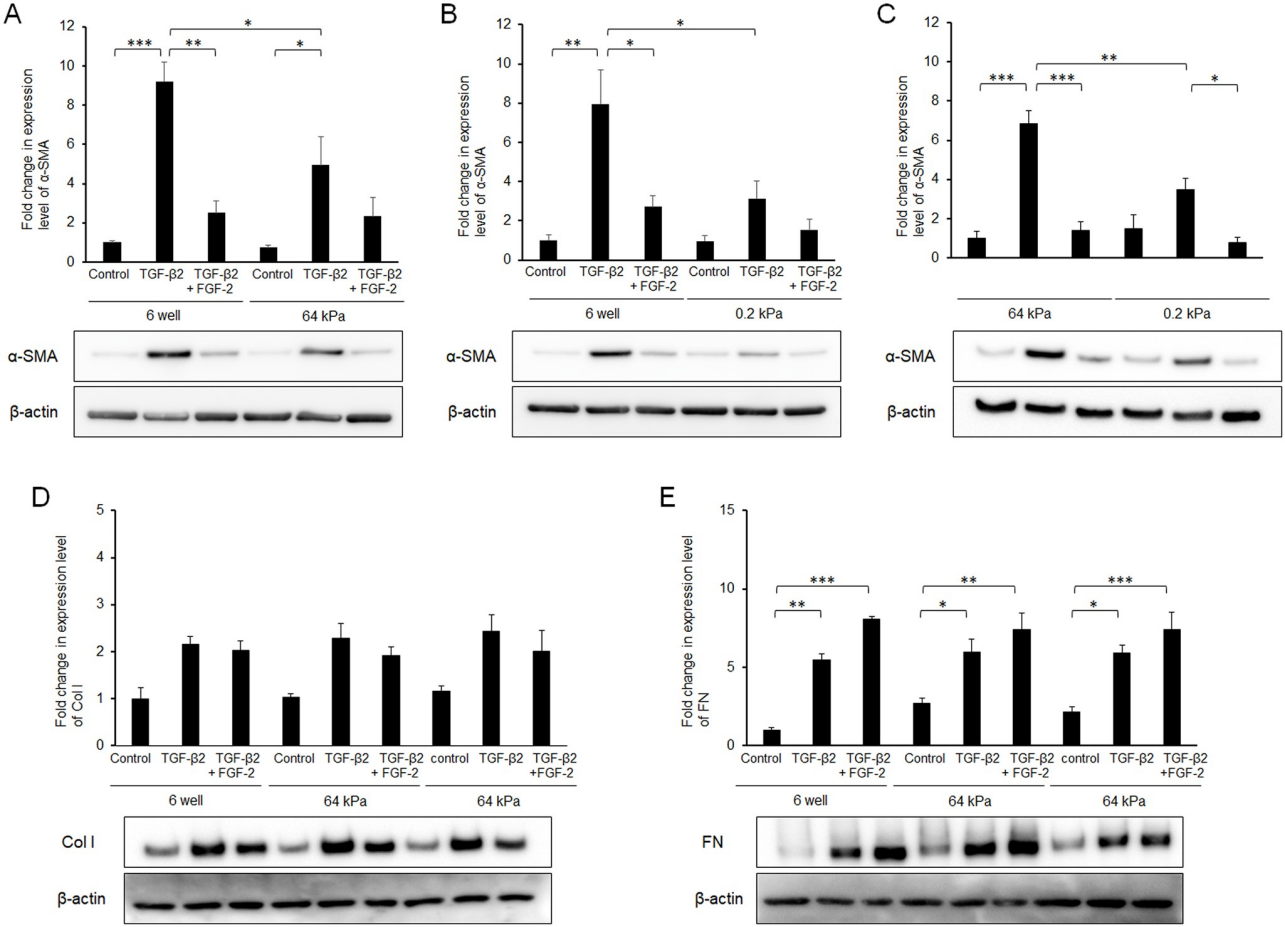
Effects of FGF-2 and/or a soft substratum on TGF-β-induced expression of α-SMA. Conjunctival fibroblasts were cultured on normal plastic wells or soft substratum wells (64 or 0.2 kPa), and treated with FGF-2 (10 ng/mL) and/or TGF-β2 (2.5 ng/mL) for 48 h, followed by Western blot analysis. The induction of α-SMA by TGF-β2 was decreased on a soft substratum in the softness-dependent manner. FGF-2 suppressed TGF-β-induced α-SMA expression even on the soft substratum. *p < 0.05. Data are shown as the mean ± SE, n = 3.

**Fig 6:**
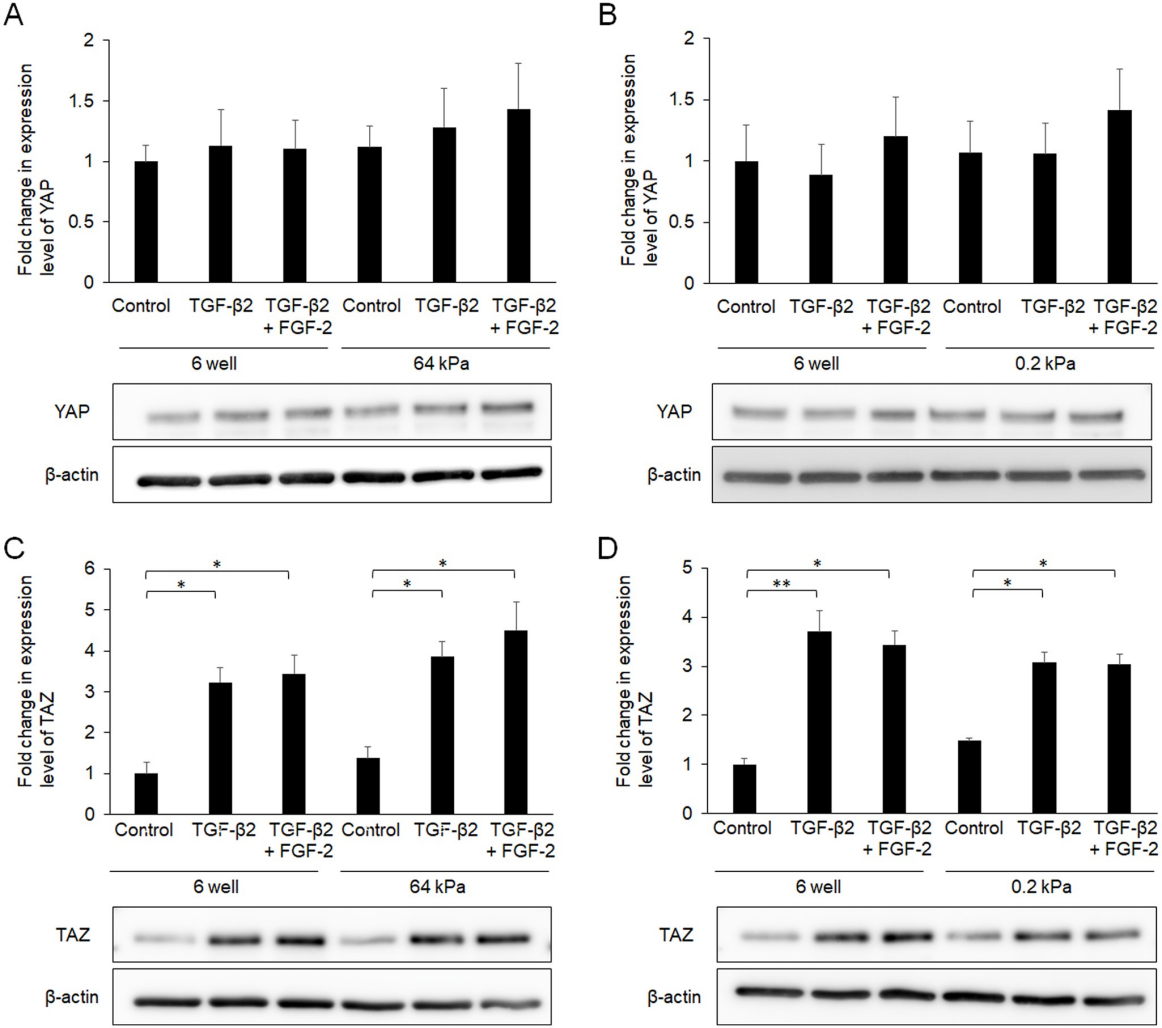
Effects of FGF-2 and/or soft substratum on the expression of YAP (A) and TAZ (B). Conjunctival fibroblasts were cultured on normal plastic wells or soft substratum wells (64 or 0.2 kPa), and treated with FGF-2 (10 ng/mL) and/or TGF-β2 (2.5 ng/mL) for 24 h, followed by Western blot analysis. The expression levels of YAP or TAZ were not affected by the stiffness of the substratum. *p < 0.05, **p < 0.01. Data are shown as the mean ± SE, n = 3.

To further characterize the relationship between FGF-2 treatment and mechanical stress, we utilized culture wells that had a soft substratum (64 or 0.2 kPa). Compared to normal plastic wells, the TGF-β2-induced increase in α-SMA expression was suppressed in a softness-dependent manner (Fig 5A and 5B). Of note, its effect was softness dependent (Fig 5C). The α-SMA expression levels were similar in the presence of TGF-β2 and FGF-2 for the entire substratum, suggesting that FGF-2 functioned independently of substratum stiffness. In contrast, substratum stiffness or FGF-2 did not affect the expression levels of Col I, FN, YAP or TAZ (Figs 5D and 5E and 6).

**Fig 7:**
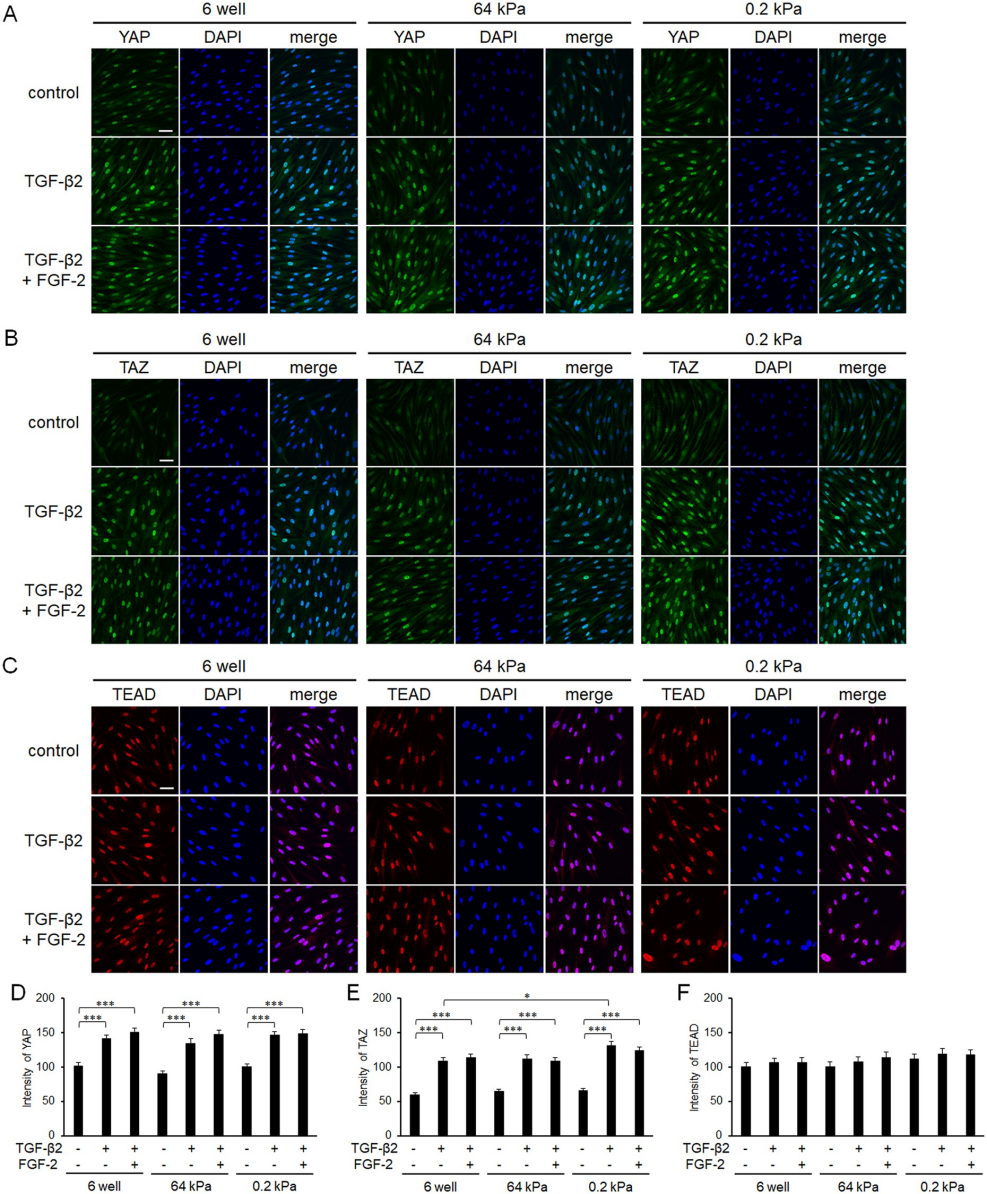
Effects of FGF-2 and/or a soft substratum on the subcellular locations of YAP, TAZ, and TEAD. Conjunctival fibroblasts were cultured on normal plastic wells or soft substratum wells (64 or 0.2 kPa), treated with FGF-2 (10 ng/mL) and/or TGF-β2 (2.5 ng/mL) for 24 h, and then subjected to immunocytochemical analysis (A-C). The nuclear localizations of the molecules were quantified based on the co-staining with DAPI (D-F). Although the nuclear levels of YAP and TAZ increased by TGF-β2 treatment, those of YAP, TAZ, and TEAD were not significantly affected by FGF-2 or substratum stiffness (n = 3). Scale bar: 50 μm.

As YAP/TAZ are active only in the nucleus, the subcellular protein locations were immunocytochemically determined. Although TGF-β2 induced nuclear translocation of YAP, neither FGF-2 nor a soft substratum had any significant effect (Fig 7). The subcellular location pattern of TAZ was similar to that of YAP. In contrast, the subcellular location of TEAD, a transcription factor that interacts with YAP/TAZ, did not change under any condition tested.

As pulmonary fibroblasts reportedly produce FGF-2 after stimulation by TGF-β1 [30], we used an ELISA to explore whether conditioned media of conjunctival fibroblasts contained FGF-2. The FGF-2 concentration was below the limit of detection (102.4 pg/mL) with or without the addition of TGF-β2; externally added FGF-2 was successfully detected.

## Discussion

In the present study, FGF-2 inhibited the TGF-β-induced expression of α-SMA and collagen type I, but not fibronectin, in conjunctival fibroblasts. Furthermore, although FGF-2 and a soft substratum did not affect TAZ induction by TGF-β2, they independently suppressed TGF-β-induced α-SMA expression. To the best of our knowledge, this is the first report to identify the relationship among TGF-β, FGF-2 and mechanical stress in conjunctival fibroblasts.

FGF-2 suppressed the TGF-β-induced induction of α-SMA in conjunctival fibroblasts, indicating that FGF-2 inhibited transdifferentiation into myofibroblasts via FGF receptors (Figs 1A and 1B and 2). In aortic valvular interstitial cells, FGF-2 reportedly repressed Smad-mediated myofibroblast activation [31]. Similarly, FGF-2 inhibited TGF-β-induced differentiation of human cardiac fibroblasts and airway smooth muscle cells into myofibroblasts [32,33]. These data accorded with the results of the present study, suggesting a general interaction among various tissues during wound healing. In contrast, the effects of FGF-2 on the expression of extracellular matrix in the present study did not correspond to the results of past reports. For example, the suppressive effect of FGF-2 on the expression of Col I was more evident in cardiac fibroblasts and dermal fibroblasts [32,34] compared to conjunctival fibroblasts (Fig 1C). Furthermore, FGF-2 did not suppress the expression of FN in the presence or absence of TGF-β2 in the present study, which differed from past reports on other tissues [34,35]. These discrepancies may be due to differences in cell origin, and/or culture conditions. FGF-2 did not affect the morphological changes induced by TGF-β2 (Fig 3B), but partially inhibited the effects of TGF-β2 on the cell properties of conjunctival fibroblasts, at least in the short term.

It is well known that FGF-2 is a mitogenic factor [36], and its effect was significant at higher concentrations in the present study (Fig 3A). As we reported previously [29], TGF-β2 also increased the proliferation of conjunctival fibroblasts by transdifferentiation into myofibroblasts. Notably, there was no additive effect of these two factors with respect to cell proliferation, suggesting that there might be a common mechanism. In past reports, TGF-β1 induced the secretion of FGF-2, thereby promoting cell proliferation in subconjunctival fibroblasts or liver fibroblasts [28,37]. Although we did not detect FGF-2 secretion in response to TGF-β2 treatment in the present study, topical elevation of the FGF-2 concentration around the cell membrane might explain the absence of additive effects on cell proliferation.

It is widely known that the transdifferentiation of fibroblasts into myofibroblasts increases contractile activity and produces more collagen, and excessive activity of myofibroblasts results in the production of stiff fibrotic tissue, which in turn causes fibrotic diseases. Recent studies have clarified that the stiff tissue itself enhances myofibroblast transdifferentiation through mechano-sensing molecules, such as YAP and TAZ [38,39]. Thus, transdifferentiation into myofibroblasts and tissue stiffness may constitute a vicious feedback cycle. Consistent with this possibility, in this study the TGF-β-induced expression of α-SMA was suppressed under soft substratum conditions (Fig 5). Notably, FGF-2 attenuated the TGF-β-induced expression of α-SMA under all substratum conditions tested, suggesting that the underlying mechanisms with respect to the suppressive effect of TGF-β2 on α-SMA expression may differ between FGF-2 treatment and the soft substratum condition.

Because we showed that YAP/TAZ were essential for TGF-β2-mediated conjunctival fibrosis in a previous study [29], we herein assessed the expression levels and subcellular locations of YAP and TAZ in the presence of FGF-2. Contrary to expectations, FGF-2 increased the expression of TAZ, while the effect on YAP expression was not significant (Fig 4), and a soft substratum did not affect YAP/TAZ expression or sufficiently to explain its anti-fibrotic effects (Fig 6). In addition, although nuclear localization of YAP/TAZ was enhanced by TGF-β2 as reported previously [29], neither FGF-2 nor a soft substratum exhibited any such effect (Fig 7). Taken together, the results show that the fibrotic changes induced by FGF-2 or the soft substratum were independent of YAP/TAZ expression and subcellular location in conjunctival fibroblasts, at least under our experimental conditions. Recently, it was reported that stiffness-dependent YAP localization is overridden by low- or high-density Matrigel (a polyacrylamide hydrogel) regardless of substrate stiffness. A low ligand concentration was associated with a low nuclear level of YAP and a high ligand concentration with a high nuclear level [40]. It was difficult to estimate ligand density on the cell surface; we therefore lack data on any such effect. Given that YAP was located in the nucleus of even cells on the soft substratum, the ligand concentration may be high under our experimental conditions. Further study is required to explore the relationship between YAP/TAZ locations and the substratum stiffness of conjunctival fibroblasts. However, we found that a soft substratum suppressed α-SMA expression without affecting YAP/TAZ activity.

In conclusion, either FGF-2 treatment or a soft substratum suppressed TGF-β-induced transdifferentiation of conjunctival fibroblasts into myofibroblasts. Although their molecular mechanisms were not identified through the analysis of YAP/TAZ expression levels or their subcellular locations, FGF-2 attenuated the TGF-β-induced expression of α-SMA, even in the soft substratum condition, suggesting that the underlying mechanisms of the suppressive effect of TGF-β2 on α-SMA expression may differ between the FGF-2 treatment and the soft substratum condition.

## Supporting information

S1 File The original uncropped and unadjusted images underlying all blot results reported. (PDF)
