# Correction: TGF-β-induced activation of conjunctival fibroblasts is modulated by FGF-2 and substratum stiffness

**DOI:** 10.1371/journal.pone.0251615

**Published:** 2021-05-06

**Authors:** Tomoyo Matsumura, Tomokazu Fujimoto, Satoshi Iraha, Akiko Futakuchi, Yuji Takihara, Fumika Watanabe-Kitamura, Eri Takahashi, Miyuki Inoue-Mochita, Hidenobu Tanihara, Toshihiro Inoue

Satoshi Iraha should be included in the author byline. Satoshi Iraha should be listed as the third author, and his affiliation is 4: Department of Ophthalmology, National Sanatorium Kikuchi Keifuen, Koshi, Kumamoto, Japan. The contributions of this author are as follows: Contributed to data correction and writing of the manuscript during revision, including conducting all quantitative RT-PCR experiments.

The correct citation is: Matsumura T, Fujimoto T, Futakuchi A, Iraha S, Takihara Y, et al. (2020) TGF-β-induced activation of conjunctival fibroblasts is modulated by FGF-2 and substratum stiffness. PLOS ONE 15(11): e0242626. https://doi.org/10.1371/journal.pone.0242626
