# Supplementary material for: TGF-β-induced activation of conjunctival fibroblasts is modulated by FGF-2 and substratum stiffness
Source: PLoS One. 2020 Nov 18;15(11):e0242626. doi: 10.1371/journal.pone.0242626 (PMC7673499; doi:10.1371/journal.pone.0242626)
Supplement: S1 File — (PDF) [file pone.0242626.s001.pdf]

Figure 1-A

used for figure

Top: α-SMA
Bottom: β-actin

①control ②10 ng/ml FGF-2 ③50 ng/ml FGF-2 ④100 ng/ml FGF-2
⑤TGF ⑥TGF+ 10 ng/ml FGF-2 ⑦TGF+ 50ng/ml FGF-2 ⑧TGF+100 ng/ml FGF-2

①②③④⑤⑥⑦⑧ ①②③④⑤⑥⑦⑧ ①②③④⑤⑥⑦⑧ ①②③④⑤⑥⑦⑧

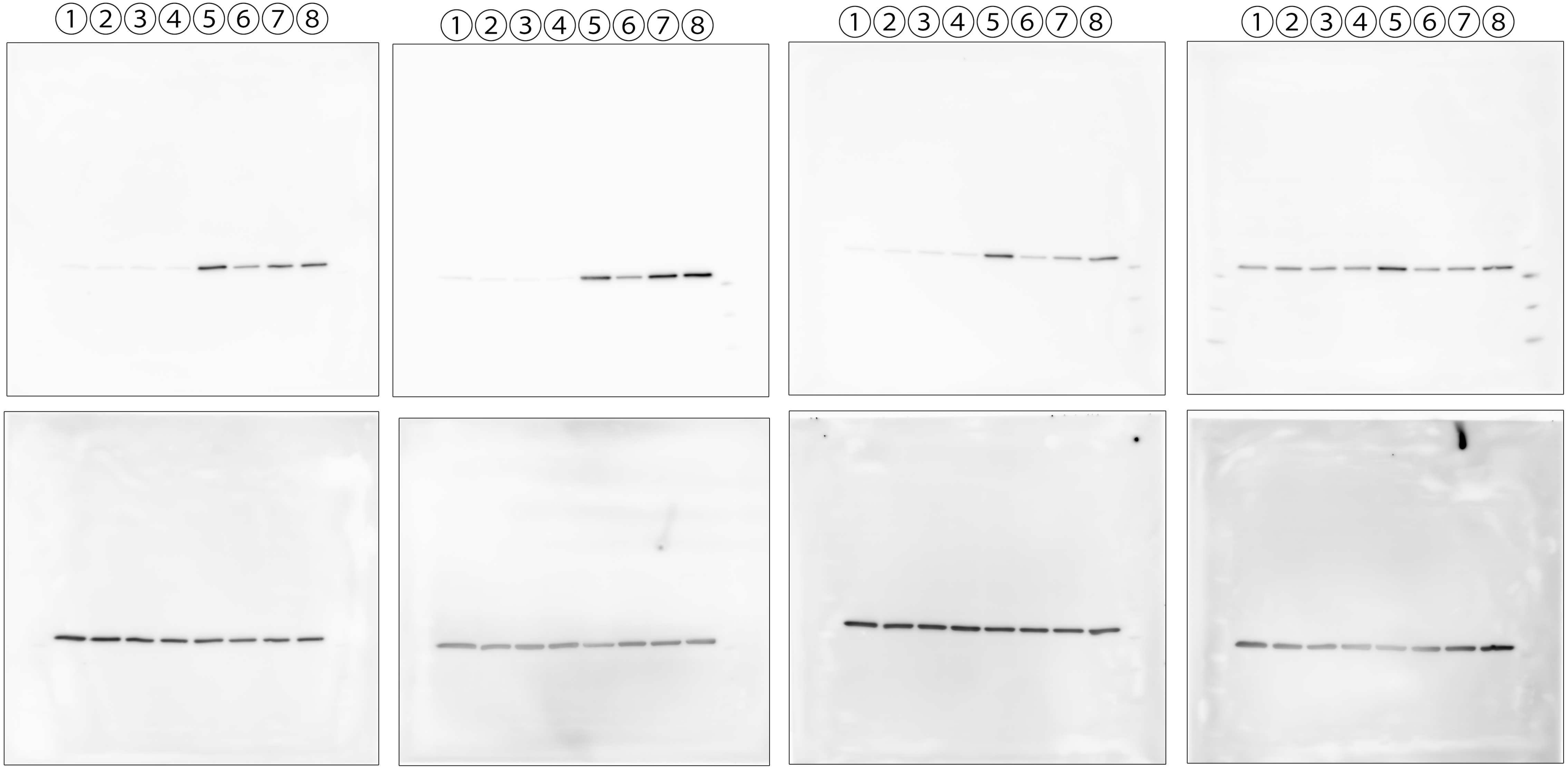

Figure 1-B

Top: α-SMA
Bottom: β-actin

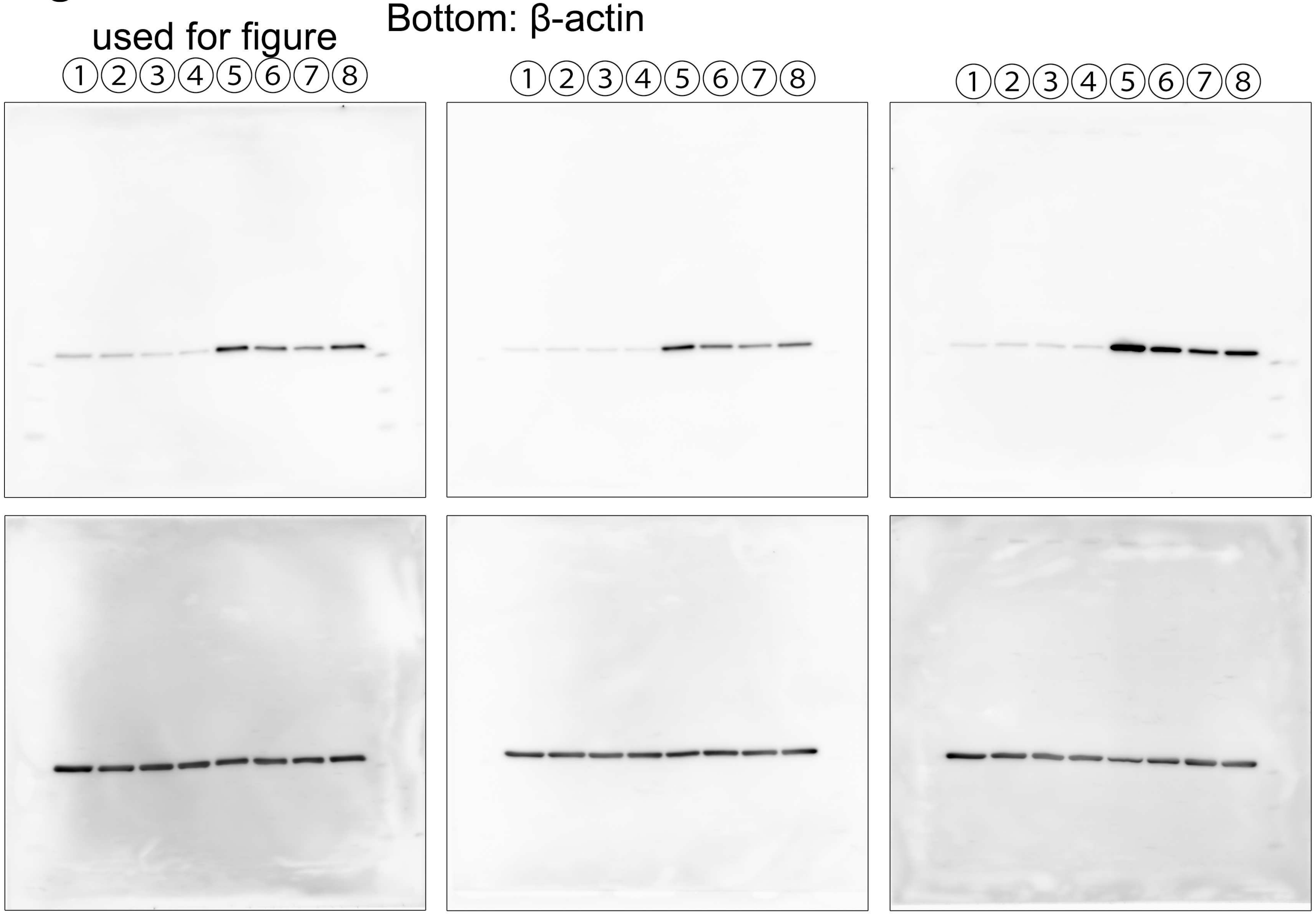

①control
②2 ng/ml FGF-2
③10 ng/ml FGF-2
④50 ng/ml FGF-2
⑤TGF ⑥TGF+ 2 ng/ml FGF-2
⑦TGF+ 10ng/ml FGF-2
⑧TGF+50 ng/ml FGF-2

Figure 1-C

TOP: α-SMA

Bottom: β-actin

used for figure

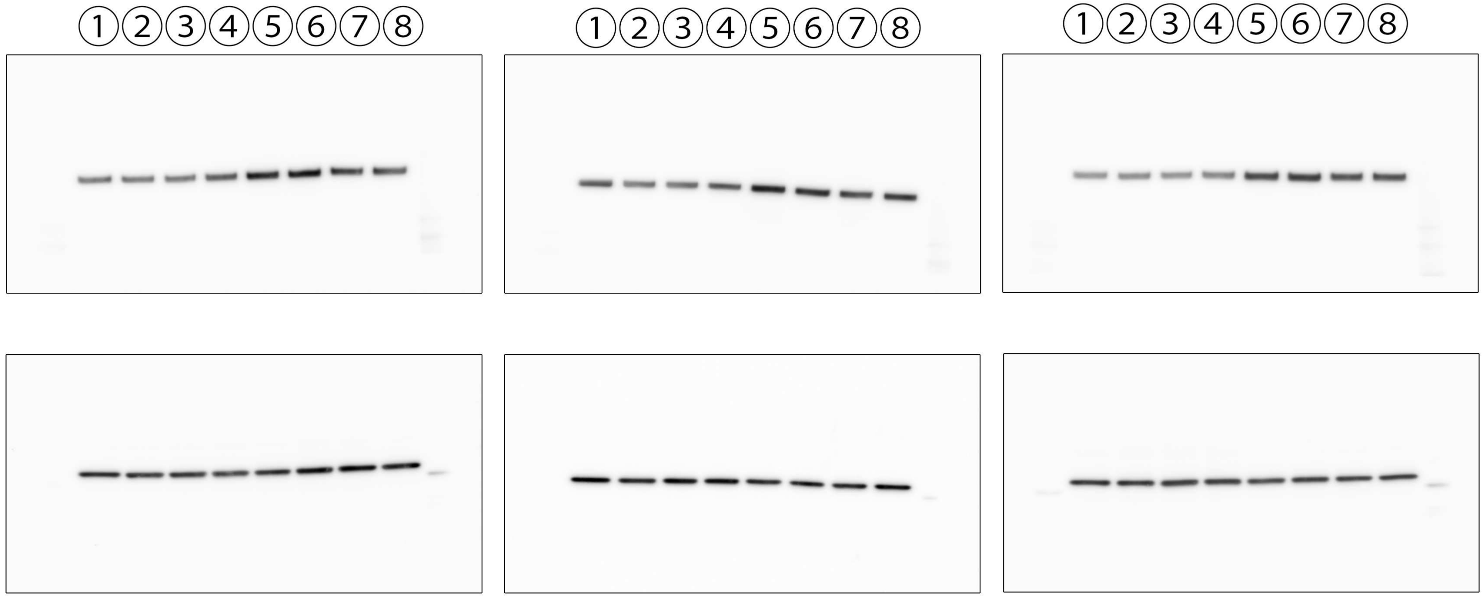

①control
②2 ng/ml FGF-2
③10 ng/ml FGF-2
④50 ng/ml FGF-2
⑤TGF
⑥TGF+ 2 ng/ml FGF-2
⑦TGF+ 10ng/ml FGF-2
⑧TGF+50 ng/ml FGF-2

Figure 1-D

Top: fibronectin
Bottom: β-actin

used for figure

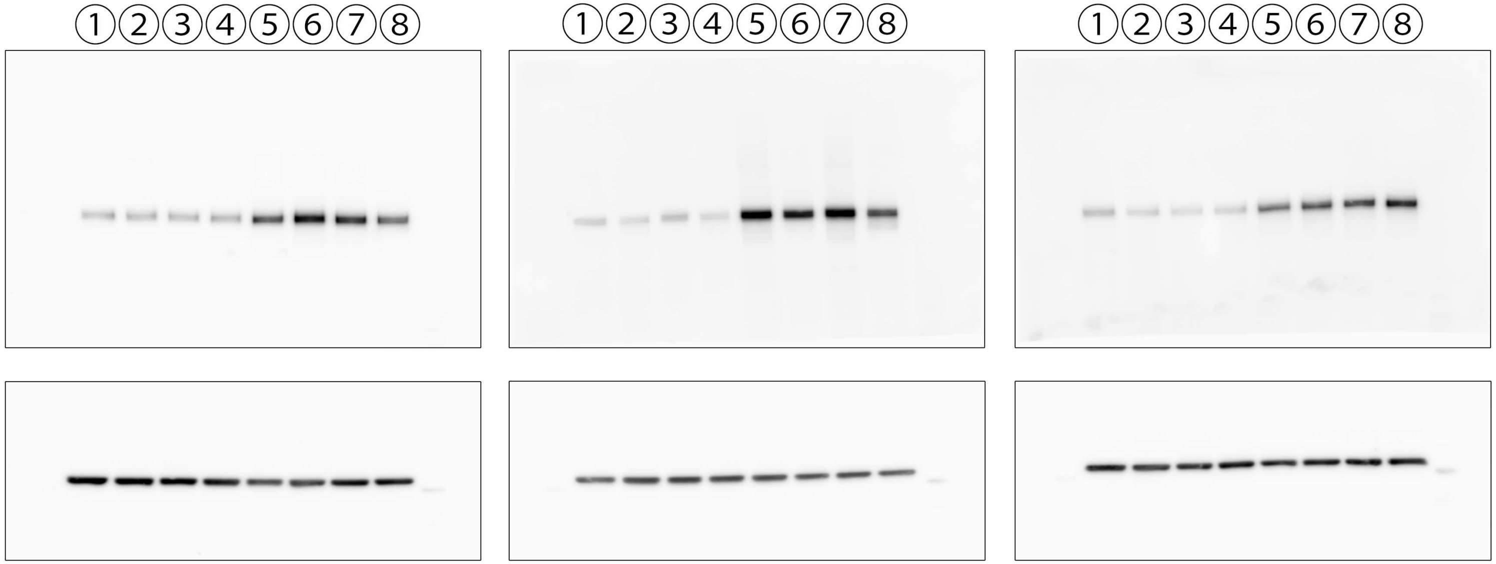

①control
②2 ng/ml FGF-2
③10 ng/ml FGF-2
④50 ng/ml FGF-2
⑤TGF
⑥TGF+ 2 ng/ml FGF-2
⑦TGF+ 10ng/ml FGF-2
⑧TGF+50 ng/ml FGF-2

Figure 2-A
Top: α-SMA
Bottom: β-actin

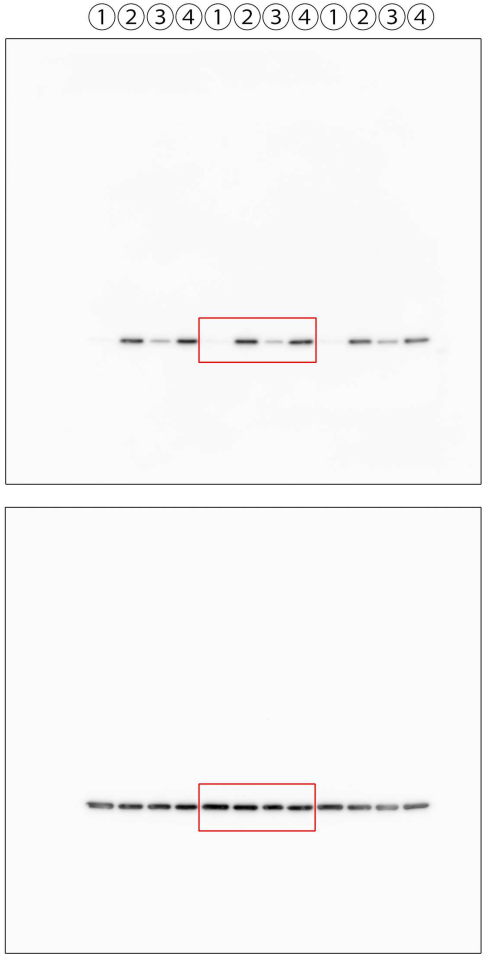

Figure 2-B
Top: COL 1
Bottom: β-actin

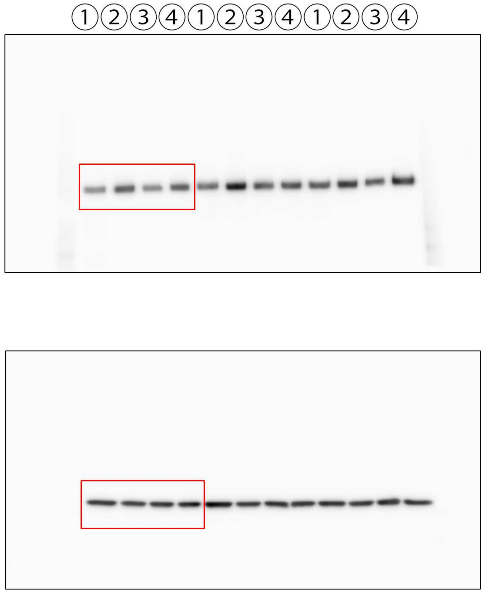

Figure 2-C
Top: fibronectin
Bottom: β-actin

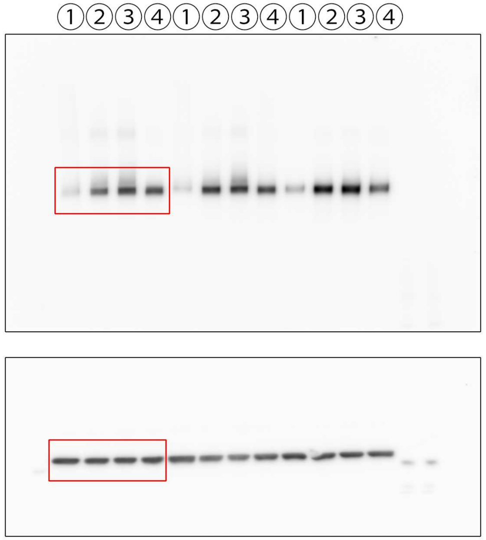

①control
②TGF
③TGF+FGF-2
④TGF+FGF-2+PD173074

Three samples were electrophoresed per gel.

The part used in the figure is shown in a red frame.

Figure 4-A

Top: YAP (70 kDa)
Bottom: β-actin

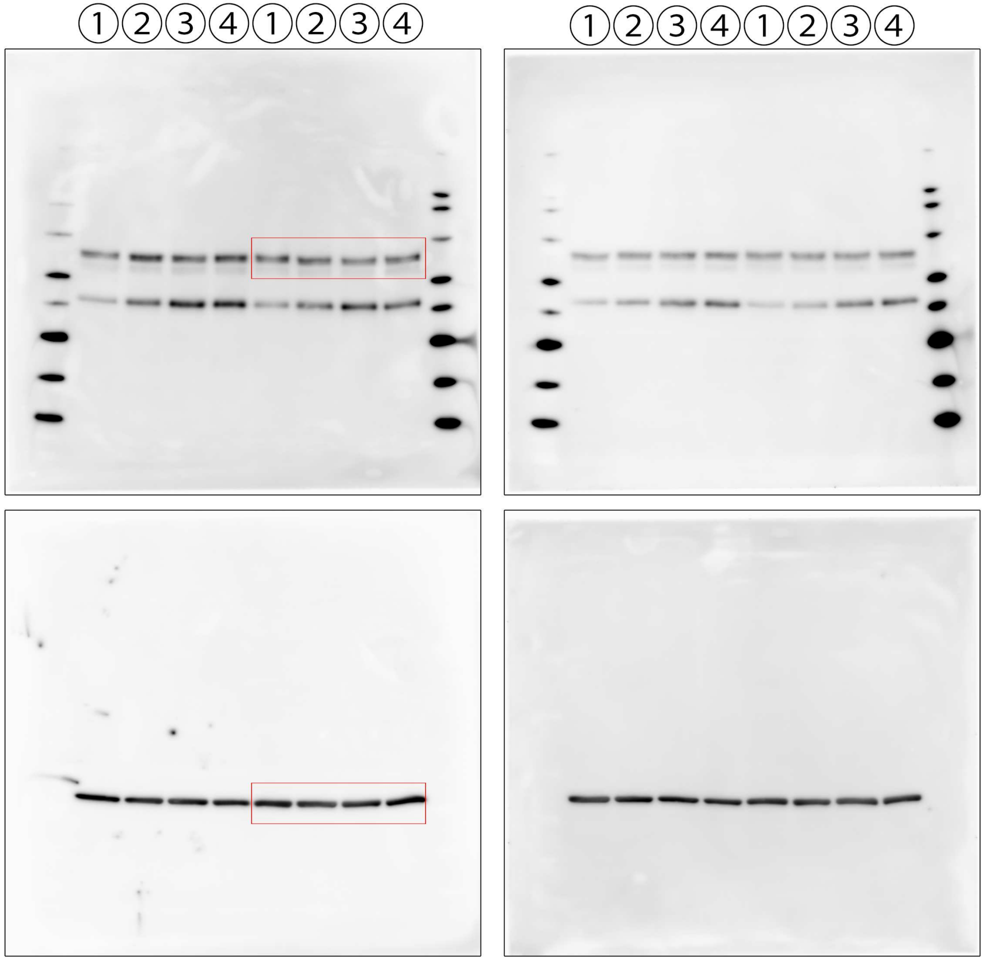

①control
②FGF-2
③TGF
④TGF+FGF-2

Two samples were electrophoresed per gel.
(4 samples in total)

The part used in the figure is shown in a red frame.

Figure 4-B

Top: TAZ
Bottom: β-actin

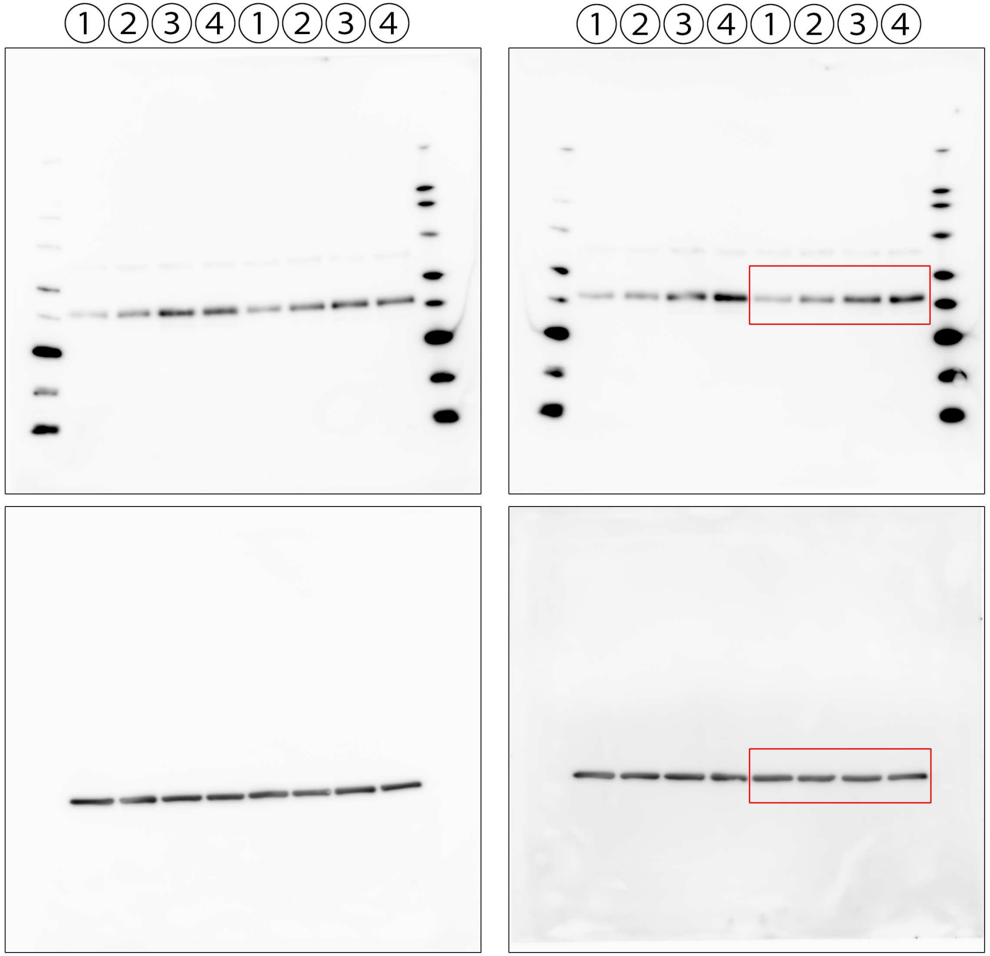

①control
②FGF-2
③TGF
④TGF+FGF-2

Two samples were electrophoresed per gel. (4 samples in total)

The part used in the figure is shown in a red frame.

Figure 5-A

Top: α-SMA
Bottom: β-actin

used for figure

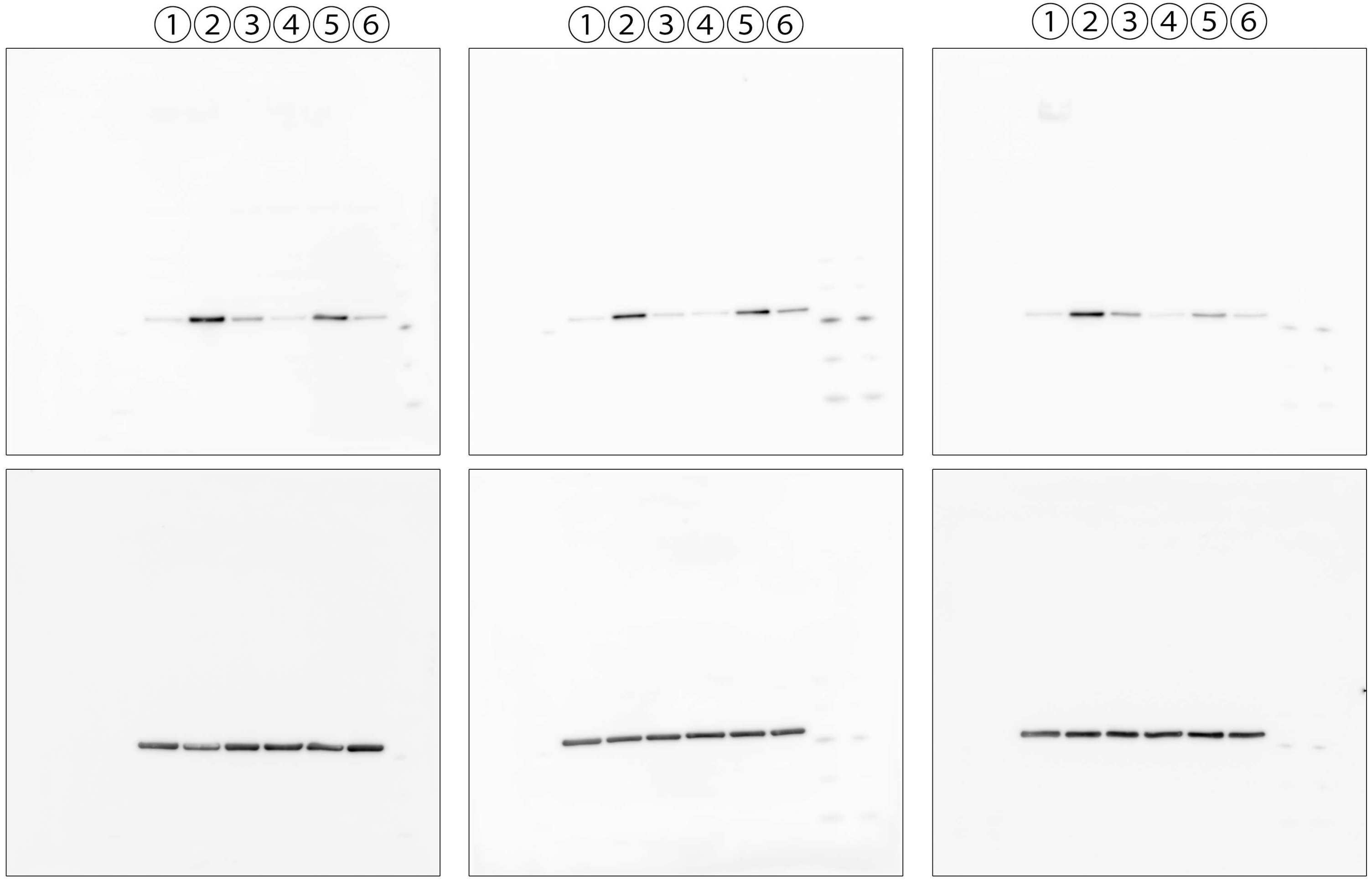

①6well-control
②6well-TGF
③6well-TGF+FGF-2
④64 kPa-control
⑤64 kPa-TGF
⑥64 kPa-TGF+FGF-2

Figure 5-B

Top: α-SMA
Bottom: β-actin

used for figure

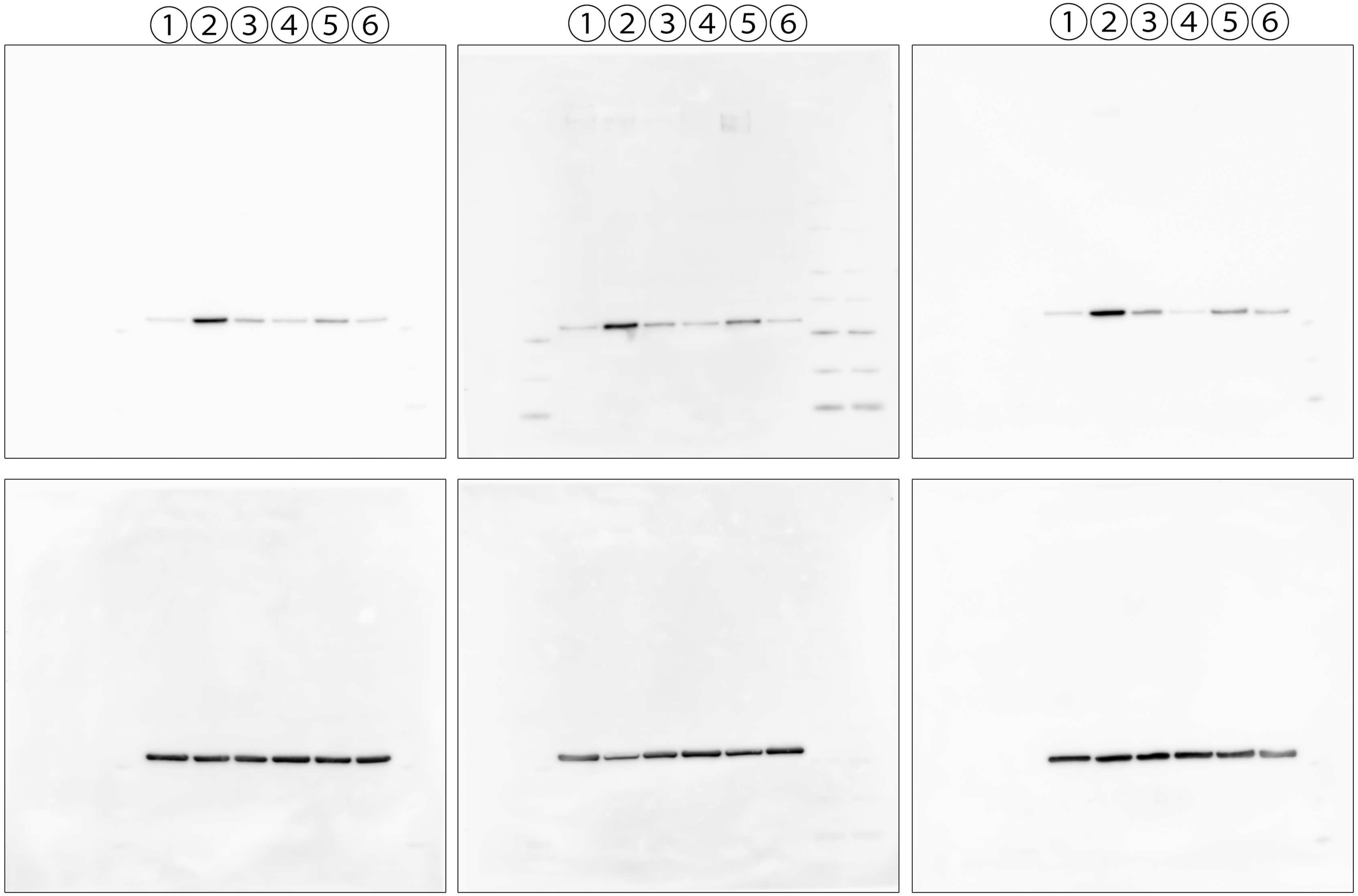

①6well-control
②6well-TGF
③6well-TGF+FGF-2
④0.2 kPa-control
⑤0.2 kPa-TGF
⑥0.2 kPa-TGF+FGF-2

Figure 5-C

Top: α-SMA
Bottom: β-actin

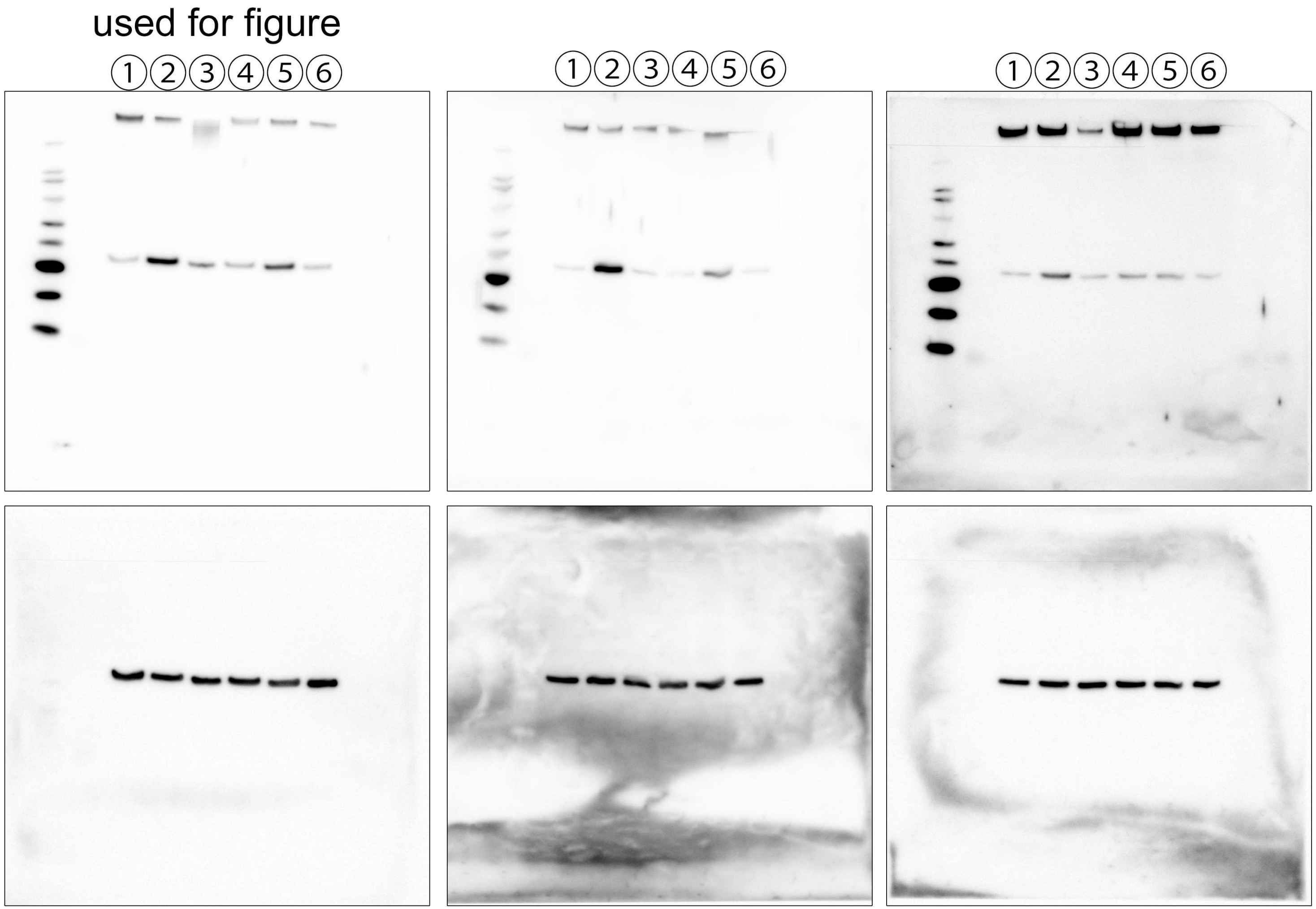

①64 kPa-control
②64 kPa-TGF
③64 kPa-TGF+FGF-2
④0.2 kPa-control
⑤0.2 kPa-TGF
⑥0.2 kPa-TGF+FGF-2

Figure 5-D

Top: Col I
Bottom: β-actin

used for figure

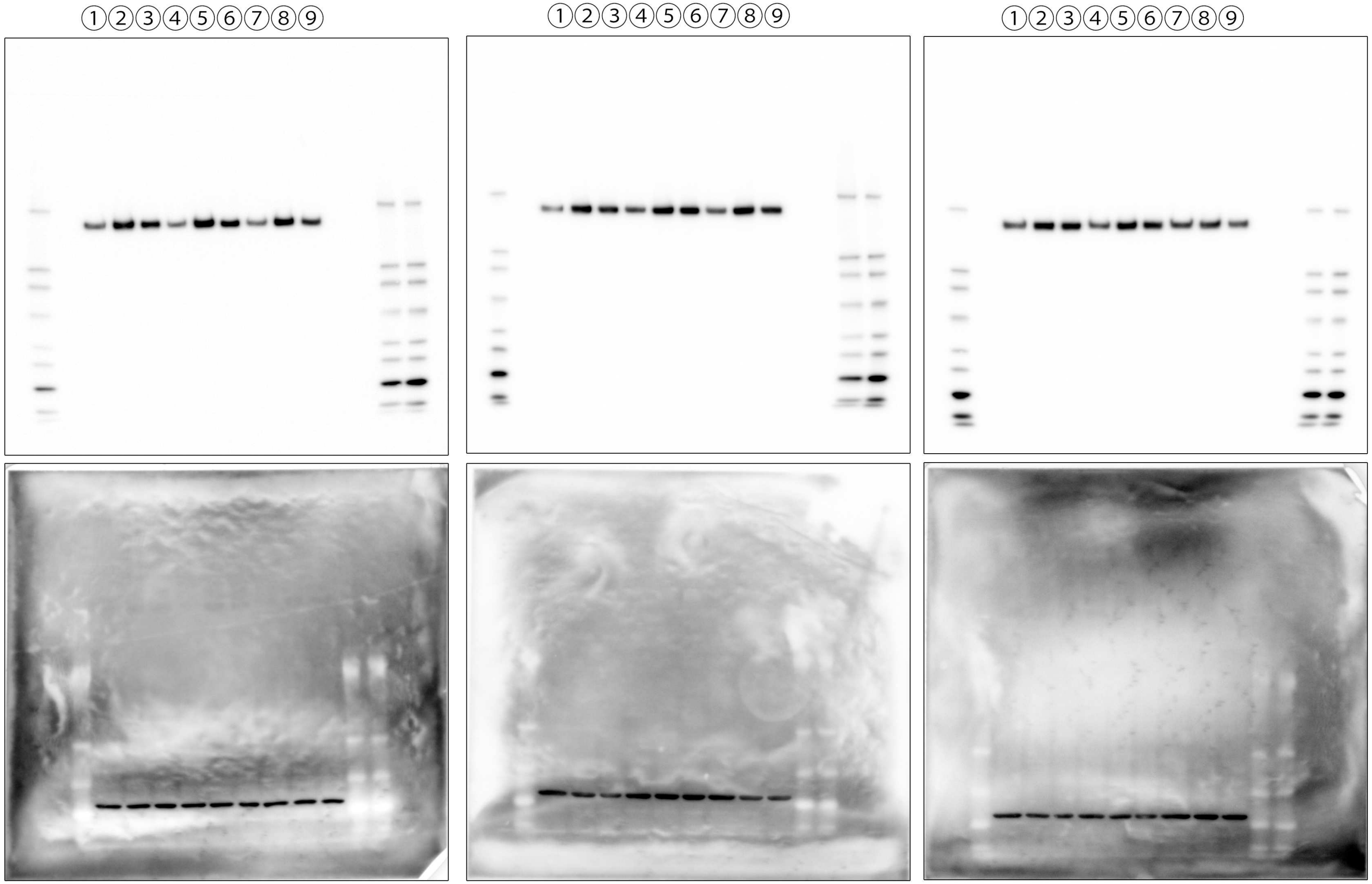

①6well-control
②6well-TGF
③6well-TGF+FGF-2
④64 kPa-control
⑤64 kPa-TGF
⑥64 kPa-TGF+FGF-2
⑦0.2 kPa-control
⑧0.2 kPa-TGF
⑨0.2 kPa-TGF+FGF-2

Figure 5-E

Top: FN
Bottom: β-actin

used for figure

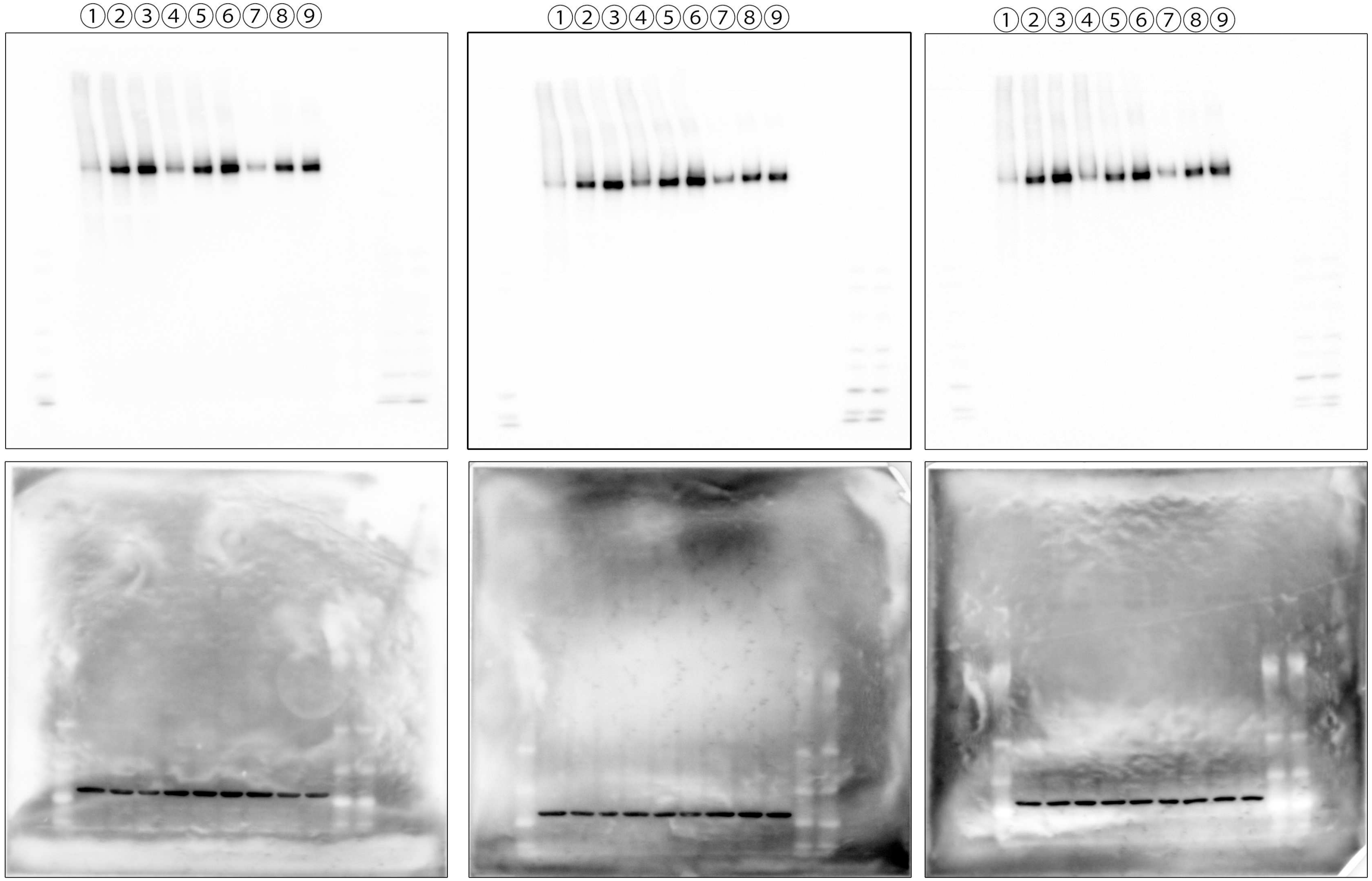

①6well-control
②6well-TGF
③6well-TGF+FGF-2
④64 kPa-control
⑤64 kPa-TGF
⑥64 kPa-TGF+FGF-2
⑦0.2 kPa-control
⑧0.2 kPa-TGF
⑨0.2 kPa-TGF+FGF-2

Figure 6-A

Top: YAP (70 kDa)
Bottom: β-actin

used for figure

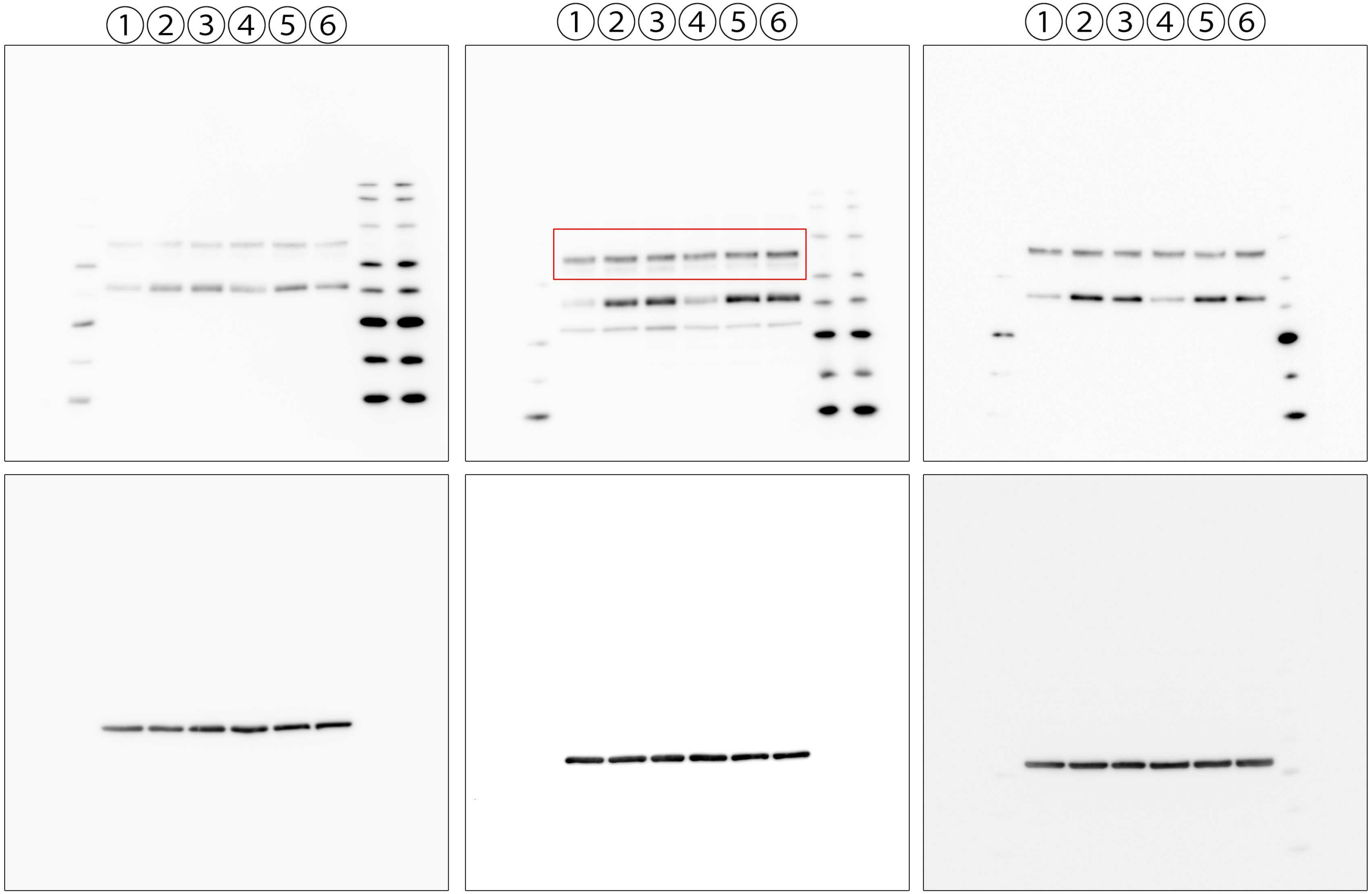

①6well-control
②6well-TGF
③6well-TGF+FGF-2
④64 kPa-control
⑤64 kPa-TGF
⑥64 kPa-TGF+FGF-2

Figure 6-B

Top: YAP (70kDa)
Bottom: β-actin

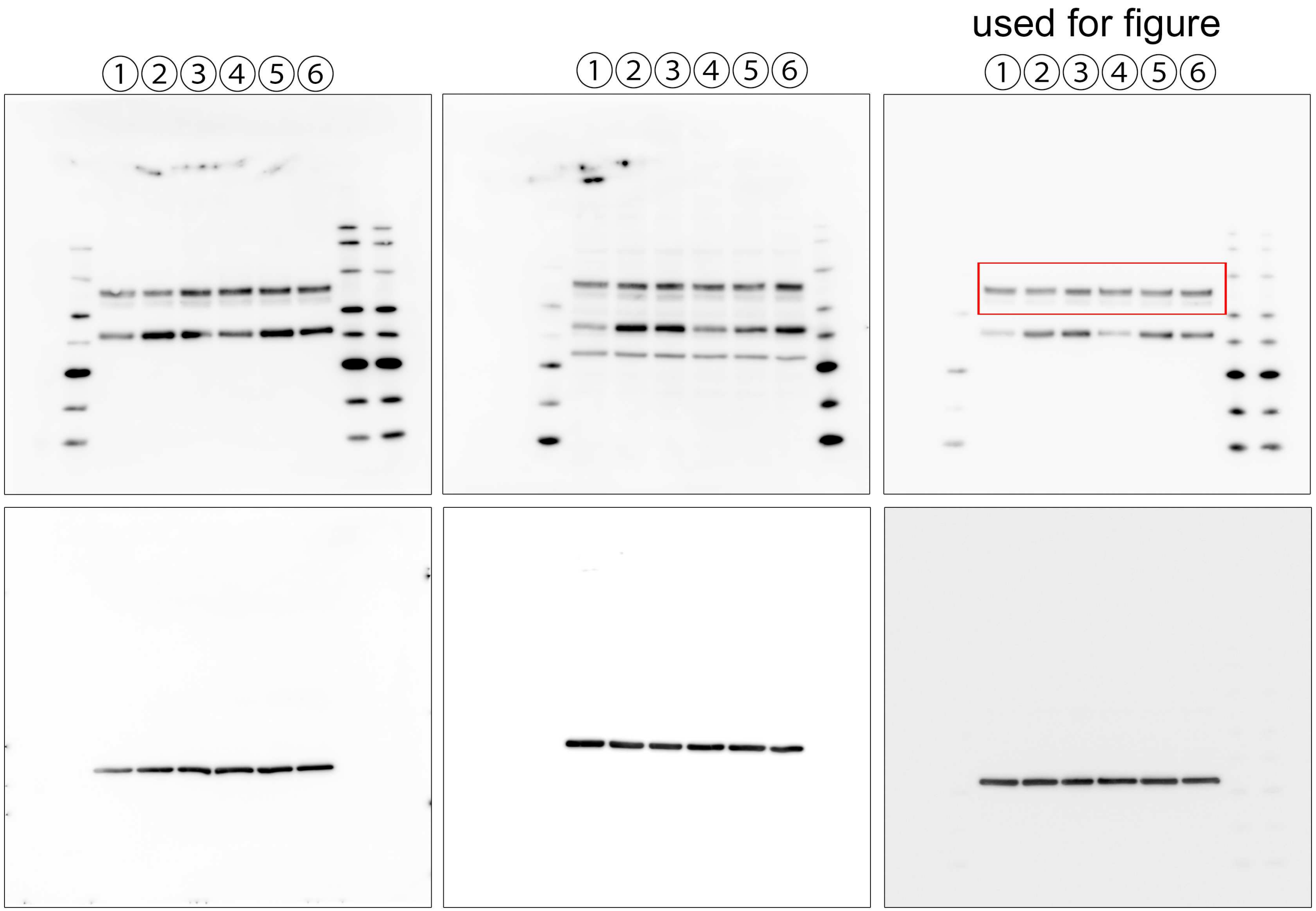

①6well-control
②6well-TGF
③6well-TGF+FGF-2
④0.2 kPa-control
⑤0.2 kPa-TGF
⑥0.2 kPa-TGF+FGF-2

Figure 6-C

Top: TAZ
Bottom: β-actin

used for figure

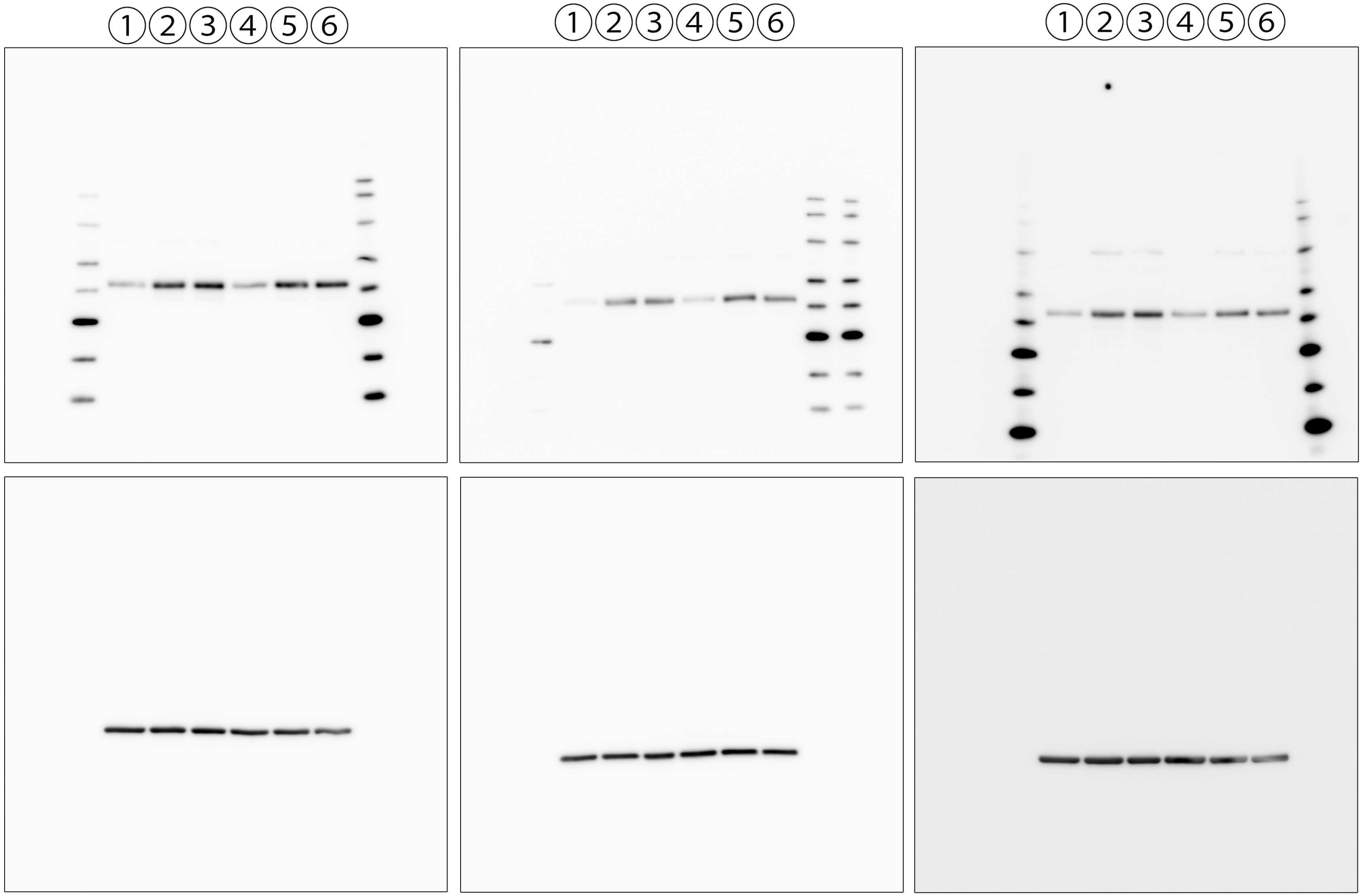

①6well-control
②6well-TGF
③6well-TGF+FGF-2
④64 kPa-control
⑤64 kPa-TGF
⑥64 kPa-TGF+FGF-2

Figure 6-D

Top: TAZ
Bottom: β-actin

used for figure

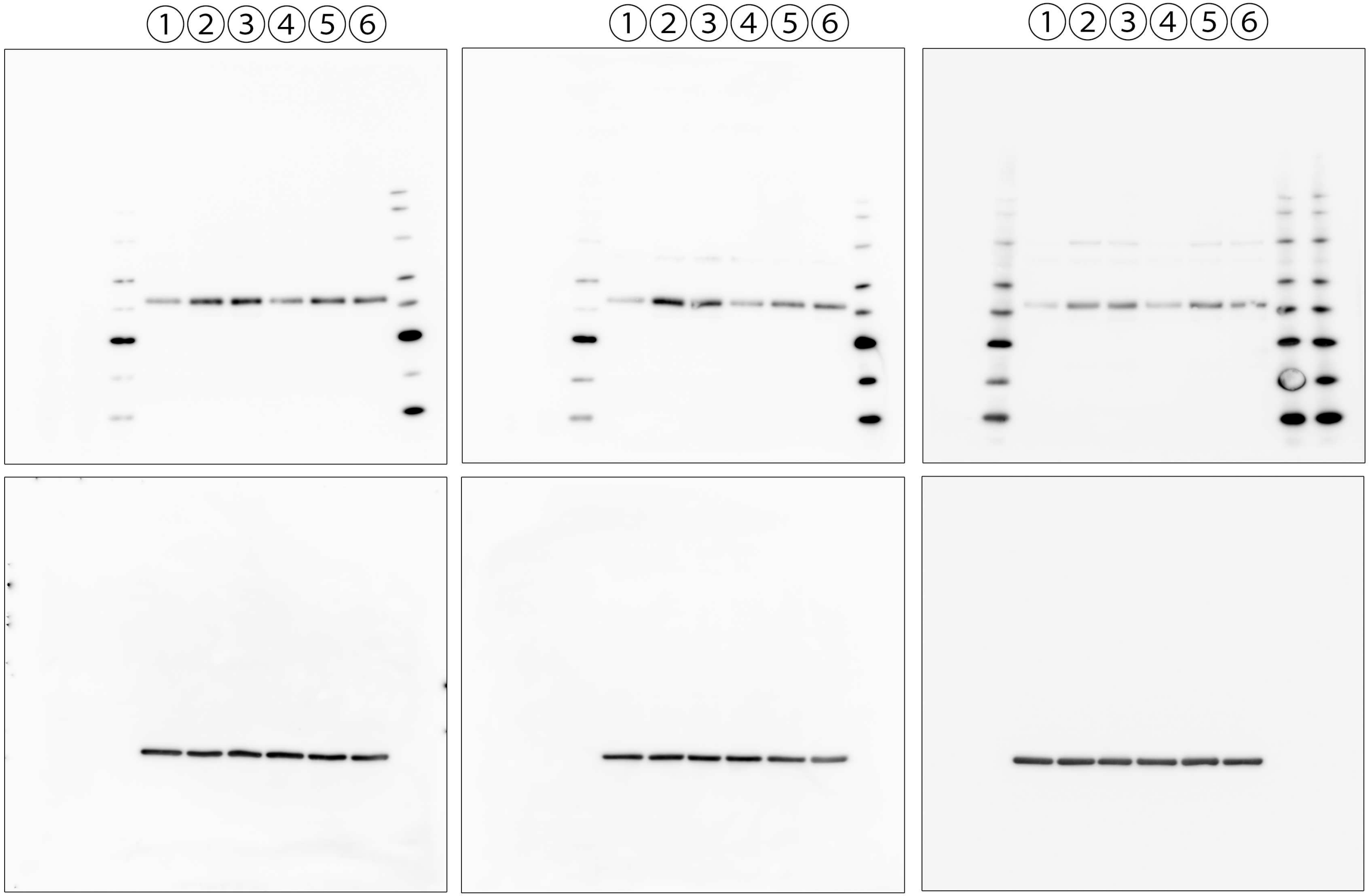

①6well-control
②6well-TGF
③6well-TGF+FGF-2
④0.2 kPa-control
⑤0.2 kPa-TGF
⑥0.2 kPa-TGF+FGF-2
